# Effects of oral glutamine supplementation on jejunal morphology, development, and amino acid profiles in male low birth weight suckling piglets

**DOI:** 10.1371/journal.pone.0267357

**Published:** 2022-04-27

**Authors:** Johannes Schregel, Johannes Schulze Holthausen, Miriama Sciascia, Zeyang Li, Solvig Görs, Anja Eggert, Armin Tuchscherer, Jürgen Zentek, Cornelia C. Metges

**Affiliations:** 1 Research Institute for Farm Animal Biology (FBN), Institute of Nutritional Physiology, Dummerstorf, Germany; 2 Freie Universität Berlin, Department of Veterinary Medicine, Institute of Animal Nutrition, Berlin, Germany; 3 Research Institute for Farm Animal Biology (FBN), Institute for Genetic and Biometry, Dummerstorf, Germany; INIA, SPAIN

## Abstract

**Background:**

It has been shown that small intestine development in low birth weight **(LBW)** piglets is impaired. Glutamine **(Gln)** has been reported to improve piglet health and intestinal function in weaned piglets, but data is scarce in suckling piglets. This study was conducted to investigate the effects of oral Gln supplementation compared to Alanine (**Ala**) on jejunal development and function in 5 and 12 d old male LBW and normal birth weight **(NBW)** suckling piglets.

**Results:**

Gln had no effect on the jejunal morphology, development, tissue and digesta amino acid profiles and mRNA abundance of genes involved in amino acid transport, metabolism, glutathione synthesis in LBW piglets when compared to Ala supplementation and birth weight controls at 5 and 12 d. Only the concentration of Gln in jejunal tissue was higher in NBW piglets supplemented with Gln compared to Ala at 5 d (P < 0.05). A comparison of the birth weight groups showed no differences between LBW and NBW piglets at 5 and 12 d in any parameter. Jejunal crypt depth, villus height / width, tunica muscularis thickness, number of goblet and IgA positive cells, the ratio of jejunal RNA to DNA and the concentration of DNA, protein and RNA changed (P < 0.05) from 5 compared to 12 d. The concentrations of several free, and protein bound amino acids as well as amino metabolites differed between age groups in jejunal tissue but the digesta concentrations were affected to a lesser extent.

**Conclusions:**

Oral Gln supplementation to suckling male piglets over the first 12 d of life was not associated with changes in jejunal parameters measured in this study. The absence of effects may indicate that Gln is absorbed as well as metabolized in the upper intestinal tract and thus could benefit intestinal development at a more proximal location.

## Introduction

Increasing litter sizes in modern pig production have led to higher numbers of LBW piglets [1]. Low birth weight is accompanied by an increased risk of disease, impaired organ development, and higher mortality [2–4]. In terms of animal welfare, the high rate of mortality in LBW piglets, especially in male piglets [5], is ethically debated [6] and results in significant economic losses [7]. The pig is additionally interesting because it is considered as an excellent animal model for human nutrition [8]. Underweight infants often have problems related to immature development of the intestinal tract [9].

The small intestine (**SI**) has digestive, absorptive as well as immunological functions and grows rapidly in the early neonatal period [10]. This rapid growth is fueled by colostrum and milk intake, which provides not only energy and essential nutrients, but also different bioactive compounds such as growth hormones [11]. The jejunum is the largest section of SI [12] and during the neonatal period the morphology, several metabolic pathways and immunological functions are constantly changing [13–15]. Previous studies in piglets show that jejunal morphology, development and function is impaired in LBW individuals [16–18]. To overcome this impairment several nutritional strategies have been developed [1, 19]. These include supplementations with colostrum [20] or bovine whey protein [21], nucleotides [22], short-chain fatty acids [23] and specific amino acids (**AA**) [24].

Glutamine **(Gln)** and glutamate (**Glu**) are the most abundant protein bound amino acids **(PBAA)**, whereas free glutamine in sow milk increases during lactation and becomes the most abundant free amino acid **(FAA)** [25]. *In vitro* studies have shown that Gln is a primary energy source for neonatal porcine enterocytes [26–28]. It is assumed that Gln promotes protein synthesis, immune response and oxidative status in mucosal cells of SI [29]. In enterocytes, Gln can be transformed to Glu, which is a precursor of glutathione, a key anti-oxidative defense molecule [30]. Gln metabolism by the jejunum has been investigated in several species, including pigs [27, 31, 32]. However, the majority of the Gln supplementation studies in pigs have been conducted in weaned piglets [29, 33–36], which are, from a physiological perspective, different from suckling piglets. In these studies Gln has been supplemented as single AA [34–36] or as dipeptide [29, 33], with most of the control groups supplemented with isonitrogenous amounts of alanine (Ala). Studies supplementing Gln to piglets during the later suckling period, analyzing parameters after weaning, have been conducted as well [33, 37–39]. Studies with lactating sows suggest that the Gln provided by milk might be limiting for protein synthesis of piglets [39]. Hence, it was also investigated whether supplementation of maternal diet with Gln either during pregnancy or lactation [40] was beneficial, but the effects on piglet growth were inconclusive.

To the best of our knowledge, this is the first study to investigate the effect of oral Gln supplementation on jejunal morphology, development and AA profiles in tissues and digesta in sow reared piglets. Since jejunal morphology as well as development is impaired in LBW compared to NBW piglets [18], we hypothesized that Gln supplementation would improve these parameters in LBW piglets compared to their Ala supplemented control littermates. In addition, changes in jejunal AA profiles could provide insights into jejunal Gln metabolism and its potential role in improving LBW jejunal morphology and development. The aim of this study was to investigate the effects of oral Gln supplementation to suckling piglets with different birth weights on jejunal characteristics including morphology, AA-metabolism and anti-oxidative-defense.

## Methods

### Animals, experimental design and sample collection

All experimental procedures were approved by the licensing authority State Office for Agriculture, Food Safety and Fishery Mecklenburg-Western Pomerania, Germany (permission No. 7221.3-1-026/16), and performed according to the German Animal Welfare Act following the Directive 2010/63/EU (European Convention for the Protection of Vertebrate Animals used for Experimental and Other Scientific Purposes). Healthy German Landrace gilts were bred and gave birth at the Research Institute for Farm Animal Biology experimental pig facility, where the entire study was conducted [41].

The trial design has been previously described in detail [42]. Briefly, male LBW with a mean birth weight **(BiW**) of 1.1 ± 0.04 kg (n = 48; below the lowest BiW quartile of the experimental pig facility) [42] and NBW (1.49 ± 0.04 kg; n = 48; represents the middle 50^th^ percentile of piglets born at the experimental pig facility) littermates selected at birth. Within 24 h post farrowing, litter sizes were standardized to 12 piglets and experimental piglets assigned to either Gln (1 g/kg BW/d; n = 48) or Ala (1.22 g/kg BW/d; isonitrogenous to Gln; n = 48) supplementation. Each LBW or NBW sibling was assigned to a supplementation (Ala, Gln) or age-group (5 or 12 d) in order to obtain similar mean birth weights of LBW (5 or 12 d; LBW-Ala vs. LBW-Gln) or NBW (5 or 12 d; NBW-Ala vs. NBW-Gln) supplementation pairings. Not more than three piglet pairs per sow were selected. Experimental piglets remained with, and were suckled by their respective dam throughout the study, which was performed across 17 experimental blocks. Approximately 24 h post birth, experimental piglets were orally supplemented with Gln or Ala as described [42]. Piglets were supplemented 3 times daily (07:00, 12:00 and 17:00) with 1/3 of the calculated daily dose using disposable syringes. The procedure used to orally dose the piglets with the supplemental amino acids is described in the S1 File. Exclusion criteria for pairs of piglets in this study were loss of body weight for more than two consecutive days, sickness or lack of mobility of already one of the paired piglets. During the experimental period 5 pairs of LBW and NBW piglets were excluded accordingly. Excluded pairs were replaced by matching pairs of piglets to reach the total sample size (n = 96). In addition, no blinding was conducted during the study, with all participants knowing the experimental group allocations from birth.

At 5 and 12 d, piglets were transported to the Research Institute for Farm Animal Biology slaughterhouse 2.5 h prior to euthanasia. Two h before euthanasia each piglet received 33% of their respective daily AA supplement in 6 mL milk replacer (150 g/L water at 45°C; 16.5 MJ of metabolizable energy (ME)/kg, 20.5% crude protein, 10% crude fat, 0.2% crude fiber; Neopigg Rescuemilk 2.0, Provimi, Netherlands). Piglets were electro-stunned and euthanized by exsanguination. Within 5 min post-euthanasia a 35 (5 d) or 40 cm (12 d) jejunal tissue section was sampled from a defined anatomical site in each animal and age group (5 d; ~40 cm, 12 d; ~60 cm prior to the ileocecal junction). Digesta was collected, snap-frozen in liquid N_2_, and stored at -80°C for subsequent analysis. The jejunal tissue was then washed with physiological saline and a 5 cm section (most proximal to the ileocecal junction) put into Roti-Histofix (4% paraformaldehyde, Histofix, Roth, Karlsruhe, Germany) for histological analysis. The remaining tissue was diced into small pieces, snap frozen in liquid nitrogen and stored at -80°C for subsequent analysis.

### Jejunal morphometry, histochemistry and immunohistochemistry

Histo-fixed jejunum samples were cut with a feather-trimming blade (FEATHER, No.130 Type(S)) into 1 large and 2 smaller pieces and prepared as previously described [43]. A microtome (Type 1400 Fa. Leitz Wetzlar, Germany) was used for cutting 5 μm sections from the paraffin blocks. For mucosal morphometry measurements and differentiation of diverse mucin types defined by the carbohydrates displayed, the Alcian blue pH 2.5 -periodic acid Schiff staining method described by Liu et al. (2014) [44], was used. The measurements were investigated using a microscope (Photomicroscope BX43F, Olympus, Tokyo, Japan) equipped with a digital camera (Olympus DP72, Tokyo, Japan). Pictures were examined with the cellSens imaging software (v. 1.4, Olympus). Five villi and corresponding crypts were randomly chosen from various well-orientated parts of at least four sections. Sections with undamaged villi and crypts were cut longitudinally. The distance from the tip of the villi to the bottom of the crypts was measured. Morphometric measurements included villus height **(VH)** (from the tip of the villus to the crypt mouth), villus width **(VW)**, crypt depth **(CD)** (from the crypt mouth to the base of the crypt), villus height to crypt depth ratio and tunica muscularis thickness **(TuM)** [44].

For quantification of Immunoglobulin-A (IgA) secreting cells, 5 μm jejunal paraffin sections were mounted on glass slides. After deparaffinization and rehydration, the slides were boiled in 0.1 M sodium citrate buffer (pH 6.0). Endogenous peroxidase was inhibited with 1% aqueous hydrogen peroxide solution for 30 min at room temperature. Slides were then incubated in a humid chamber for 1 h in PBS containing 10% normal horse serum to avoid nonspecific antibody binding. Afterwards, sections were incubated over night at 4°C with the following antibody: goat anti-porcine IgA 1:4000 (NB724, Novus Biologicals, Abingdon, UK). Subsequently washed sections were incubated for 1 h with biotinylated horse anti goat IgG 1:500 (Cat. NO: BA-9500, Vector Laboratories) and then administered with ABC complex (Vectastain elite ABC peroxidase Kit, Standard, Vector Laboratories). To visualize the immune reaction, a 3,3´-diaminobenzidine chromogen solution (DAB Substrate kit, Vector Laboratories) was applied [45]. An isotype control with a non-specific antibody (goat IgG, AB-108-c, R&D Systems) was conducted to avoid nonspecific binding of the Fc part of the primary antibody. IgA positive cells were counted in the jejunal lamina propria in three areas in five eye fields from four sections per animal according to Waly et al. (2001) [46]. The areas were delineated with cellSens imaging software (v. 1.4, Olympus), ignoring the epithelium, large blood vessels and artefacts. In each area, stained cells were counted and the results were given as positive cells per 10,000 μm^2^ of lamina propria tissue [47].

**Fig 1:**
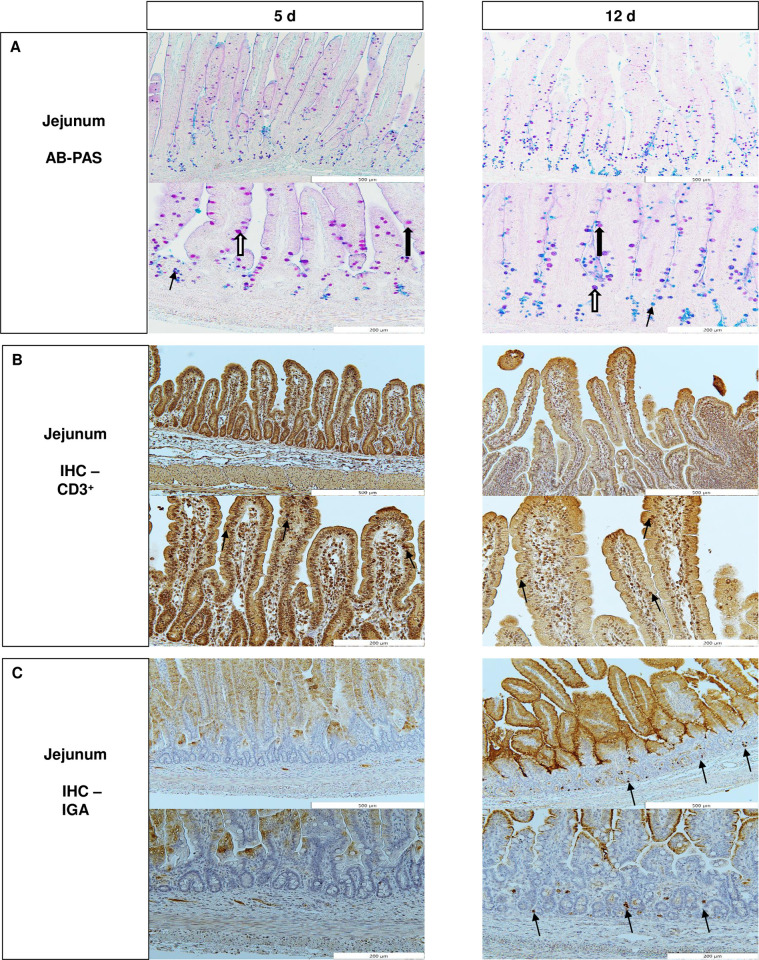
Jejunal Histomorphology and Immunohistochemistry (IHC) of 5 d and 12 d old male suckling piglets. **A** Alcian blue pH 2.5 -periodic acid Schiff stained jejunal tissue with stained goblet cells, different arrows indicating goblet cells containing different mucins. Narrow arrow = acidic mucins, wide closed arrow = neutral mucins, wide open arrow = mixed mucins. 5 d, 12 d: upper picture 100 x, lower picture 200 x magnification. **B** IHC of CD3, arrows indicating positive stained intraepithelial CD3+ cells in villi. 5 d, 12 d: upper picture 100 x, lower picture 200 x magnification. **C** IHC of IgA positive stained cells in lamina propria, no IgA positive cells detected at day 5, arrows indicating IgA positive cells. 5 d, 12 d: upper picture 100 x, lower picture 200 x magnification. Villus tip stained probably by milk derived secretory IgA (SIgA) on the apical side of the enterocytes.

The detection of CD3-positive intraepithelial lymphocytes was performed as described previously [48]. Briefly, for antigen retrieval, slides were heated in 0.1 M sodium citrate buffer (pH 6.0) in a microwave oven until boiling for 30 min. Afterwards the primary antibody PPT3 (mouse anti porcine CD3 epsilon, CAT NO 4510–01, Southern Biotech) was applied to the slices. An isotype control with a non-specific antibody (mouse IgG, CAT NO 0102–01, Southern Biotech) was included, to control nonspecific binding of the Fc part of the primary antibody. For visualization of the primary antibody, a two-step indirect method was used (mouse and rabbit Specific HRP/DAB IHC Detection Kit, ab236466, ABCAM). The secondary antibody was conjugated with horseradish peroxidase (HRP) labelled micro-polymer (goat anti-rabbit HRP Conjugate, 58009 ABCAM). The whole immunohistochemistry protocol was performed according to a published procedure [49]. To evaluate the stained sample a double-blind quantification of CD3-positive intraepithelial lymphocytes was performed. Only complete and intact villi (two slices per animal, five villi per slice) were evaluated and cell counts were expressed per 100 enterocytes (Fig 1).

### Free and protein bound AA and AA metabolites concentration in jejunal tissue and digesta

Jejunal tissue samples were prepared as previously described [50] and tenfold diluted by ultrapure water for FAA analysis. For the assessment of total amino acids **(TAA)**, an enzymatic hydrolysis was performed [51]. Two μL of supernatant was diluted with 55 μL HEPES buffer (50 mM, pH 7.5), combined with 1 μL of pronase E (54 Units/mL) (Sigma-Aldrich, Munich, Germany), 1 μL of prolidase (250 Units/mL) (Sigma-Aldrich, Munich, Germany), and 1 μL of aminopeptidase M (25 Units/mL) (MP Biomedicals, Santa Ana, California) and incubated at 37°C for 20 h. Samples were centrifuged (4°C) at 16,000 *g* for 10 min, and diluted 15/100 by ultrapure water. Digesta samples were lyophilized and 5 mg was suspended in 500 μL of ultrapure water. Samples were vortexed for 15 s and centrifuged at 17,000 *g*, 4°C, for 10 min. The supernatant was transferred to a new test tube without the lipid layer and diluted by factor 4 with ultrapure water for FAA determination. For the assessment of TAA, samples were hydrolyzed enzymatically as described above with the exception that 20 μL of supernatant and 37 μL HEPES buffer were used. Free AAs, AA metabolites and TAA were measured by HPLC as described earlier [52] using 5 μm C18 columns, 250 x 4 mm HyperClone^TM^ 120 Å or 250 x 4.6 mm Gemini® 110 Å (both Phenomenex, Aschaffenburg, Germany). Protein bound AA were calculated by subtracting FAA from TAA concentrations.

### Jejunal biochemical indices and fractional protein synthesis rate

Total RNA and DNA was extracted from ground jejunal tissue (80–120 mg) according to the manufacturer’s protocol (peqGOLD TriFast; VWR International GmbH, Hannover, Germany), whereas total protein was isolated, using a lysis buffer [50] described above. Total RNA and DNA was quantified using a Nanophotometer (Implen GmbH, Munich, Germany), whilst total protein was quantified photometrically using BCA reagent (Biorad Laboratories, Feldkirchen, Germany). Biochemical indices of cell size (protein:DNA ratio), protein synthetic efficiency (protein:RNA ratio) and protein synthetic capacity (RNA:DNA ratio) were calculated as previously described [53].

Fractional protein synthesis rate **(FPSR)** was determined using the flooding dose method as described [54] with modifications. Piglets were given an intraperitoneal injection of L-^2^H_5_ phenylalanine (Ring-^2^H_5_, 99.1% atom ^2^H; ^2^H_5_-Phe; Euriso-Top, Saint-Aubin, France) (125 mg/kg BW) in physiological saline (Serumwerk Bernburg AG, Bernburg, Germany) one h before euthanasia, to measure the jejunal FPSR.

Fifty mg of ground jejunal tissue was suspended in 0.5 mL of 0.2 M perchloric acid kept on ice, using a sonication tip (Amplitude 80, cycle 0.5, 30 pulses), vortexed and centrifuged (4°C) at 3,000 *g* for 10 min. The FAA containing supernatant was adjusted to pH 7 using 4 M KOH. After centrifugation (4°C) at 3,000 *g* for 10 min, the supernatant was dried at 60°C under nitrogen. Samples were treated with N-Methyl-N-tert-butyldimethylsilyltrifluoroacetamide to form tert-butyldimethylsilyl-derivatives. Additionally, the protein pellet was used to determine the protein-bound ^2^H_5_-Phe enrichment. After washing the protein pellet twice with 1 mL of 0.2 M perchloric acid and with 1 mL of ultrapure water it was dried at 60°C under nitrogen gas. The dried pellet was hydrolyzed as described [55] and the free AA were converted to tert-butyldimethylsilyl-derivatives. The abundance of ^2^H_5_-Phe was quantified using GC-MS (Quadrupole, GC-MS QP 2010, Shimadzu, Japan, equipped with a Zebron ZB-5HT column, 30 m × 0.25 mm × 0.25 μm column, Phenomenex, USA) as described [55]. The diagnostic ions m/z 336 (M+0) and m/z 341 (M+5) were used to calculate the enrichment as molar per cent excess of ^2^H_5_-Phe. The FPSR was calculated using the following equation:


$$FPSR(\%/d)=\frac{E_{Protein}}{E_{free}}\times \frac{1}{t}\times 100$$

Where E_Protein_ is the enrichment of ^2^H_5_-Phe in the jejunal tissue protein and E_free_ is the enrichment of ^2^H_5_-Phe in the free AA pool of the jejunal tissue at the time of sampling. The period between ^2^H_5_-Phe injection and sampling is defined as t. The FPSR is expressed as the percentage of tissue protein renewed per d (%/d).

### Jejunal transcript abundances related to AA transport, AA metabolism and antioxidative defense

#### Purification of RNA and cDNA synthesis.

Total jejunal RNA (30 μg) extracted for the calculation of biochemical indices was purified using RNeasy minikits (Qiagen, Hilden, Germany) and quantified using a Nanophotometer (Implen GmbH, Munich, Germany). The RNA quality was assessed using a Bioanalyzer 2100 and RNA 6000 Nano kit (Agilent Technologies, Waldbronn, Germany), with an RNA integrity number range of 6.9 and 9.7 (mean 8.8 ± 0.8). Purified RNA (500 ng) was reverse transcribed to make cDNA using the SensiFAST™ cDNA Synthesis Kit (Bioline, Berlin, Germany) according to the manufacturer’s instructions.

#### Primer design, real time PCR assay and data preparation.

Primers were made by Integrated DNA Technologies (IDT, Antwerp, Belgium), and selected from previous studies or designed using the IDT RealTime qPCR Assay design tool. Primers were tested using serial dilutions (1/25, 1/50, and 1/100 diluted cDNA). Due to varying mRNA abundances between targets either 1/25 or 1/50 dilutions were used for quantification. Primer details are presented in (S1 Table in S1 File). Amplified cDNA samples were analyzed on 96 well plates (Roche) using the LC 96 system (Roche Diagnostics, Mannheim, Germany). Samples were analyzed in duplicate (plus five additional samples: two inter-run calibrators, a no-template and a no-enzyme control and a water control). Quantitative real time PCR was performed using the SensiFAST SYBR No-Rox Mix (Code: 98050, Bioline, Berlin, Germany), with the template (4 μM) and all reagents at half of the manufacturers recommended volume. The same reaction conditions; enzyme activation and initial denaturation (95°C for 30 s); denaturation/annealing repeated 40 cycles (95°C for 30 s, 60°C for 20 s); and melting curve analysis from 65 to 98°C with 1°C increment every 5 s) were used for all mRNA targets analyzed. The PCR efficiency and quantification cycle values were then obtained for each sample using LinRegPCR v 2014.5 [56]. Average PCR-efficiency and quantification cycle values are reported in S1 Table in S1 File. The GeNorm applet from qBASEplus selected the reference genes from six candidates (5 d: beta actin (ACTB), ribosomal protein S18 (RPS18), and DNA topoisomerase 2-beta (TOP2B), 12 d: peptidylprolyl isomerase A (PPIA) and ribosomal protein S18 (RPS18)) as the most stably expressed across the BiW and Suppl used in this study. Reference genes were used to normalize target gene mRNA abundance in the qBASEplus software and the Cq-values were converted into log transformed calibrated normalized relative quantities (Log-CNRQ) values, taking into account amplification efficiencies, inter-run variations, and normalization factors. All data was reported as per the Minimum Information for Publication of Quantitative Real-Time PCR Experiments (MIQE) guidelines [57].

### Data and statistical analysis

The required experimental sample size (*n*) was calculated per 2 and 3 level factor combination of (1) birth weight (LBW, NBW), (2) supplementation (Ala, Gln) and (3) treatment duration / age group (5 d and 12 d), using CADEMO for Windows ANOV version 4.03 (2000; BioMath GmbH, Rostock, Germany), and the settings α = 0.05, β = 0.20. The primary outcome measures used to determine *n* were body weight gain and changes in intestinal villus height and abundance of mRNA molecules associated with oxidative status.

Normal distribution was assessed via Shapiro-Wilks criteria, followed by a linear mixed model analysis which was conducted for each of the 143 variables using the MIXED procedure of SAS (version 9.4; SAS Institute Inc., Cary, NC, USA) with three fixed factors: (1) birth weight (LBW, NBW), (2) supplementation (Ala, Gln) and (3) age group (5 d and 12 d). Unless otherwise indicated, the group size for each analysis performed was *n* = 12. Deviating group sizes are reflected in the footnotes of the corresponding table. Sow was defined as a random factor which allowed explicit modelling of the non-independence of littermates from the same sow and improved inference about the fixed effects. ANOVA F-tests for the three fixed effects and their interactions were carried out and the Tukey-Kramer test was applied to compare groups and correct for multiple testing. Least squares means **(LSM)** and their standard errors **(SE)** are reported, with the largest SE shown. Differences were considered significant if Tukey-Kramer test was *P* ≤ 0.05.

The linear mixed model analysis revealed that the factor ‘age group’ had a significant effect on the analyzed set of variables. To identify the variables discriminating the two age groups (5 d and 12 d), the N-integration with Projection to Latent Structures models with Discriminant Analysis (PLS-DA) was applied, using R 4.1.0 (R Core Team, 2021) and the mixOmics package (v6.14.1; [58]). Here, so called ‘blocks’ of variables measured on the same samples are integrated in a holistic supervised analysis. In this study, all 143 variables were first analyzed together. Cross-validation was used to evaluate the performance of the PLS-DA model, with a 10-fold cross-validation and 1000 repeats to get an accurate estimations of the error rates. Centroid distance was chosen as it is regarded a suitable measure for the complex classification problems [58]. The quality of the PLS-DA model was verified by fold cross-validation using two performance indicators: Q^2^, “goodness of prediction”, or predicted variation and R^2^, known as the goodness of fit [59]. All 143 variables were then assigned to ten individual blocks; morphology characteristics (n = 5), cell types (n = 13), biochemical indices (n = 7), mRNA target-molecules (n = 21), jejunal tissue (free AA; n = 20, AA-metabolites; n = 10, PBAA; n = 20) and digesta (free AA; n = 20, AA-metabolites; n = 7, PBAA; n = 20). Sample plots for each ‘block’ of variables are presented only to visualize the potential discriminatory ability of each component in the space spanned by the first two latent variables.

A volcano plot was generated using R 4.1.0 (R Core Team, 2021) and the effsize package (v0.8.1; [60]) and qvalue package (v2.22.0; [61]). Effect sizes (Cohen’s d) were calculated for each variable based on the estimated least square means of 5 d versus 12 d. The list of p-values of the age group fixed effect estimate was then used in conjunction with a standard false discovery rate (FDR) estimation procedure to find the number of variables to be declared as different while controlling FDR at a specified level of 0.05. The FDR-adjusted p values were calculated using the Benjamini & Hochberg procedure [62].

## Results

### Jejunal morphology and abundance of goblet cells, intraepithelial lymphocytes, and IgA positive cells

The VH was affected by Suppl (*P* = 0.041), whereas VW (*P* = 0.012) was influenced by BiW (Table 1). Age affected VH (*P* = 0.019), VW (*P* = 0.019), TuM (*P* = 0.020) and CD (*P* < 0.001). The CD in the jejunum was higher at 12 d than at 5 d in LBW-Ala piglets (*P* < 0.001) (Table 1). Mixed mucins containing goblet cells in villi were affected by Suppl (*P* = 0.007) (Table 2). Age affected the number of mixed mucins containing goblet cells in crypts (*P* = 0.025) and villi (*P* = 0.020), as well as the total number of goblet cells in the crypt (*P* = 0.029). We could not observe IgA positive stained cells in the jejunal lamina propria of 5 d old piglets. In 12 d old piglets supplementation influenced the number of IgA positive cells in the lamina propria next to the crypts (Area 3) (*P* = 0.048) (S2 Table in S1 File). The number of intraepithelial lymphocytes CD3^+^ cells in the jejunal villi (S3 Table in S1 File) and the number of CD3^+^ cells in crypt area did not differ among groups.

**Table 1:** Jejunal morphology characteristics in low and normal birth weight male suckling piglets.

		Ala		Gln			*P* values^1^		
Item	Age (d)	LBW	NBW	LBW	NBW	SE	BiW	Suppl	Age
Villus height (μm)	5	812	718	789	872	65.3	0.265	0.041	0.019
	12	899	855	1061	935	66.5			
Villus width (μm)	5	95.3	97.8	95.3	101	2.87	0.012	0.256	0.019
	12	99.9	105	103	107	2.93			
Crypt depth (μm)	5	113^e^	126	124	125	5.90	0.949	0.703	<0.001
	12	150^f^	145	149	142	5.90			
Villus height to crypt depth ratio	5	7.25	5.90	6.67	7.12	0.51	0.274	0.098	0.622
	12	6.17	6.00	7.11	6.78	0.52			
Tunica muscularis thickness (μm) ^2^	5	107	105	122	102	12.3	0.814	0.882	0.020
	12	127	144	128	126	12.2			

^e,f^ Labeled LSM within a column within one Suppl and BiW group without a common letter differ, *P* < 0.05 (Tukey-Kramer test).

Values are LSM ± SE, the largest SE is shown; n = 12/group (5, 12 d).

^1^ ANOVA F test. None of the interactions of the fixed factors (Suppl x BiW; Suppl x Age; BiW x Age or Suppl x BiW x Age) were significant (*P* > 0.05).

^2^ Tunica muscularis was damaged due to the thawing procedure. Therefore group size deviated from *n* = 12 for the parameter Tunica muscularis thickness. 5 d LBW-Ala, 5 d LBW-Gln, 12 d NBW-Gln, 12 d LBW-Gln, and 12 d NBW-Ala *n* = 11.

**Table 2:** Number of jejunal goblet cells in low and normal birth weight male suckling piglets.

			Ala		Gln			*P* values^1^
Item^2^		Age (d)	LBW	NBW	LBW	NBW	SE	Age
Villus	Acid	5	3.97	4.23	4.65	4.83	0.59	0.588
		12	4.83	4.92	4.7.0	4.38	0.60	
	Neu	5	6.58	8.24	6.76	7.02	0.72	0.901
		12	6.71	7.41	7.07	7.10	0.74	
	NA	5	7.31	8.52	5.96	6.10	0.64	0.020
		12	5.52	6.14	5.11	5.51	0.65	
	Total	5	17.8	21.0	17.4	18.0	1.38	0.318
		12	17.1	18.5	16.9	17.0	1.41	
Crypt	Acid	5	21.7	20.0	22.2	24.5	1.85	0.081
		12	18.2	18.4	19.0	21.9	1.87	
	Neu	5	12.0	13.6	12.9	11.6	1.74	0.755
		12	11.7	13.8	12.3	14.2	1.77	
	NA	5	22.6	21.6	21.6	20.5	1.58	0.025
		12	18.3	19.0	17.9	19.3	1.59	
	Total	5	56.5	55.3	56.5	56.5	2.9	0.029
		12	48.2	51.1	49.1	55.2	2.92	

Values are LSM ± SE of goblet cells containing different mucins per 1 mm basement membrane, the largest SE is shown; n = 12/group (5, 12 d).

^1^ ANOVA F test. Suppl had a significant effect on NA mucins in villus (*P* < 0.01); neither the fixed factor (BiW) nor the interactions of the fixed factors (Suppl x BiW; Suppl x Age; BiW x Age or Suppl x BiW x Age) were significant (*P* > 0.05).

^2^Acid = acidic mucins; NA = mixed neutral and acidic mucins; Neu = neutral mucins.

### Jejunal free AA and AA metabolite concentrations

There was an effect of Suppl on Gln, of BiW on Cys, and of Age on the concentration of all FAA measured in the jejunal tissue (S4 Table in S1 File), with the exception of the AA metabolites citrulline, ornithine and taurine (S5 Table in S1 File). The interaction BiW x Suppl affected α-Aminoadipic acid **(Aad)**, whereas the interaction BiW x Suppl x Age was significant for Asp, Gln, His, Ile, Met, Ser, Thr, Val, the branched-chained AA, and the Aad concentration. The concentration of Gln was higher in 5 d NBW-Gln compared to NBW-Ala piglets (*P* = 0.044). The concentration of hydroxyproline (P = 0.029) was higher in all four 5 d groups than in the 12 d groups. Higher concentrations of Aad and Ala (*P* < 0.028) were found in the jejunal tissue of 5 d group compared to 12 d group with exception of NBW-Ala. The jejunal concentrations of Ser (*P* = 0.006), Gln, His, Ile, Thr (*P* = 0.041), and the group of dispensable AAs (*P* = 0.009) were higher in 5 d compared to 12 d LBW-Ala piglets. The concentrations of Glu and 3-Methylhistidine (*P* = 0.042) were higher in 5 d compared to 12 d LBW-Gln piglets. In NBW piglets supplemented with Gln the concentrations of Gln (*P* = 0.007), Asp, Glu, Ser, the group of dispensable AA (*P* = 0.029) were higher at 5 d compared to 12 d. In 12 d LBW-Gln (*P* = 0.010) and NBW-Ala (*P* < 0.001), the jejunal Gly concentration was higher compared to 5 d.

**Fig 2:**
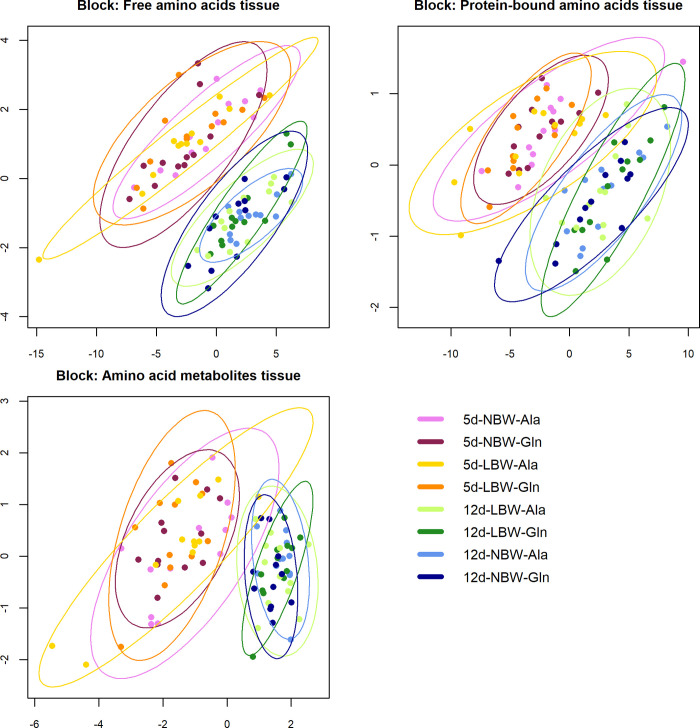
Partial Least-Squares Discriminant Analysis (PLS-DA). Sample plots of the block PLS-DA of all 143 jejunal variables assigned to ten variable groups (‘blocks’) and measured in 96 samples. Shown are the sample plots for the three blocks with the best discriminatory ability: Free amino acids tissue, Protein-bound amino acids tissue, Amino acid metabolites tissue. The other seven blocks are shown in the supplementary material (S1 Fig in S1 File). The colours indicate the eight experimental groups of the 3-factorial crossed design (birth weight: LBW/NBW, supplementation: Ala/Gln and age group: 5 d/12 d) and highlight the main comparison of the two age groups (reddish: 5 d; bluegreen: 12 d).

**Fig 3:**
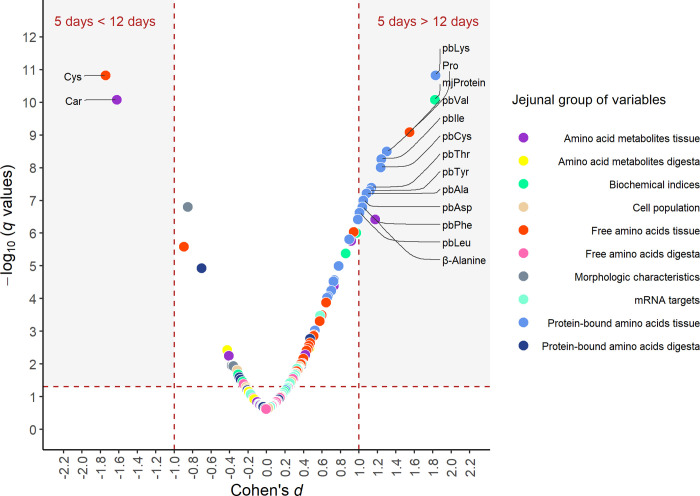
Volcano plot of jejunal variables analysed between 5 and 12 d old suckling piglets. Comparison of quantities of all 143 variables measured in 5 d and 12 d old suckling piglets. Q-values estimating the false discovery rate (FDR) were calculated for each variable from p-values of multiple Tukey-Kramer-tests comparing 5 d and 12 d old piglets. Effect sizes (Cohen’s d) were calculated for each variable based on the differences in estimated marginal means and standard deviations of 5 d versus 12 d old piglets. Differences are classified as being substantial (grey shaded area) if FDR is limited to 0.05 (q < 0.05) and if the effect size of Cohen’s d < 1 (5 d is smaller than 12 d) or Cohen’s d > 1 (5 d is larger than 12 d). The 16 variables meeting this condition are annotated.

The Block PLS-DA showed a separation between the blocks jejunal FAA and AA metabolites (Fig 2B and 2C), probably contributing to the 5 and 12 d group separation observed in the PLS-DA analysis of all experimental blocks (Fig 2; 1 comp, R^2^ = 0.65, Q^2^ = 0.65). A subsequent univariate analysis (volcano plot; Fig 3) showed that four jejunal FAA and AA metabolites were different between the two age groups (Cohen’s d ≥ 1, FDR ≤ 0.05). The FAA in jejunal tissue Pro and the AA metabolite βAla were lower in 12 d compared to 5 d age groups, whereas the Cys and the AA metabolite Car were higher in the 12 d compared to the 5 d age groups (Fig 3) (S6 Table in S1 File).

### Jejunal protein bound AA concentrations

There was an effect of Age on the concentration of all PBAAs (except Pro), whereas BiW affected Asn and Ile concentrations. The concentration of protein bound Glu, Ser (*P* = 0.049), the group of indispensable AA, branched-chained AA, dispensable AA and the group of total AA (*P* = 0.029) were higher in all 4 groups of 5 d piglets compared to the groups of 12 d piglets (S7 Table in S1 File). The concentration of protein bound Asn and Met (*P* = 0.031) was higher in groups of 5 d piglets compared to the respective groups of 12 d piglets except for the group of NBW-Ala. Higher concentrations of protein bound Arg and Trp (*P* < 0.047) were found in the jejunal tissue of the 5 d group in LBW-Ala and NBW-Gln compared to the respective 12 d groups. Lower concentrations of protein bound Arg and Trp (*P* < 0.029), were found in 12 d compared to 5 d LBW-Ala piglets. The concentrations of Gly (*P* < 0.048) were lower in 12 d compared to 5 d NBW-Gln piglets.

Block PLS-DA indicated that jejunal PBAA (Fig 2D) may be contributing to the 5 and 12 d group separation observed in the PLS-DA analysis of all experimental blocks (Fig 2A). A subsequent univariate analysis (volcano plot) showed that 10 variables (jejunal protein-bound Ile, Leu, Lys, Phe, Thr, Val, Ala, Asp, Cys, and Tyr) were lower in 12 than in the 5 d age groups (Cohen’s d ≥ 1, FDR ≤ 0.05), (Fig 3) (S6 Table in S1 File).

### Free and protein bound AA concentrations in jejunal digesta

The concentration of the digesta γ-aminobutyric acid was influenced by Suppl, whereas the FAAs Asp and Ser were affected by BiW, while Aad, α-aminobutyric acid, and Orn were affected by Age. The interaction BiW x Suppl was significant for digesta free Glu, α-aminobutyric acid while the interaction Age x Suppl affected Arg and Asp (S8 and S9 Tables in S1 File). The concentration of digesta free Aad was lower in NBW-Gln (*P* < 0.001) at 5 d compared to 12 d, and in LBW-Gln compared to NBW-Gln piglets at 5 d (*P* = 0.008). Additionally, the concentration of free Aad was higher in NBW-Gln than in NBW-Ala at 5 d (*P* = 0.006). Age was significant for the concentration of the digesta PBAAs Lys, Gln, and Pro (S10 Table in S1 File). The concentration of protein-bound Lys increased from 5 d to 12 d, in NBW-Ala piglets (*P* = 0.017).

### Jejunal biochemical indices and fractional protein synthesis

Protein, RNA, DNA (*P* < 0.001) concentrations and the RNA/DNA ratio (*P* < 0.044), reflecting protein synthetic capacity, in jejunal tissue were affected by Age. The concentration of DNA (*P* = 0.005) and RNA (*P* = 0.050) was higher in 5 d compared to 12 d LBW-Gln, NBW-Ala and NBW-Gln piglets (Table 3). Jejunal FPSR was unaffected by any of the main factors (Table 3). Subsequent univariate analysis (volcano plot) showed that the variable protein concentration was lower (FDR *P* < 0.001) in the 12 d compared to 5 d age group (Fig 3) (S6 Table in S1 File).

**Table 3:** Jejunal biochemical indices in low and normal birth weight male suckling piglets.

		Ala		Gln			*P* values^1^
Item	Age (d)	LBW	NBW	LBW	NBW	SE	Age
DNA (μg/mg _FM__^2^_)	5	4.38	4.90^e^	4.89^e^	5.17^e^	0.30	<0.001
	12	3.74	3.27^f^	3.23^f^	3.09^f^	0.31	
Protein (μg/ mg _FM__^2^_)	5	117^e^	1084	119^e^	114^e^	3.27	<0.001
	12	81.9^f^	83.4^f^	86.7^f^	84.0^f^	3.28	
RNA (μg/ mg _FM__^2^_)	5	3.97	4.41^e^	4.15^e^	3.99^e^	0.18	<0.001
	12	3.23	3.24^f^	3.10^f^	3.16^f^	0.19	
RNA/DNA	5	0.94	0.92	0.86	0.79	0.11	0.044
	12	0.97	1.07	1.13	1.13	0.11	
Protein/RNA	5	29.9	25.0	29.6	29.7	1.53	0.196
	12	25.9	26.3	28.6	27.5	1.53	
Protein/DNA	5	27.6	22.8	25.5	22.7	3.09	0.085
	12	25.9	28.9	31.8	29.5	3.12	
FPSR (%/d)^3^	5	60.7	69.8	72.8	71.6	7.42	0.123
	12	55.7	61.7	57.8	58.7	7.56	

^e,f^ Labeled LSM within a column between one Suppl—birth weight group without a common letter differ, *P* < 0.05 (Tukey-Kramer test).

Values are LSM ± SE, the largest SE is shown; *n* = 12/group (5, 12 d).

^1^ ANOVA F test. None of the other fixed factors (Suppl or BiW) or interactions of the fixed factors (Suppl x BiW; Suppl x Age; BiW x Age or Suppl x BiW x Age) were significant (*P* > 0.05).

^2^ FM = Fresh matter

^3^ Because of an insufficient accumulation of ^2^H_5_-Phe in jejunal tissue, the group size deviated from *n* = 12 for jejunal FPSR. 5 d LBW-Gln, 12 d NBW-Gln, 12 d LBW-Gln *n* = 11.

### Jejunal transcript abundance related to AA transport, AA metabolism and antioxidative defense

The BiW class affected AST-2 (*P* = 0.020) whereas Age influenced the mRNA abundance of solute carrier family 1 member 5 (SLC1A5), solute carrier family 1 member 4 variant 1 (SLC1A4V1), aspartate aminotransferase 2 (AST-2), Glu cysteine ligase (GCL), glutathione synthetase (GSS) (*P* < 0.05), and succinate dehydrogenase complex, subunit A (SDHA) (*P* < 0.001) (S11 Table in S1 File). The BiW x Age interaction affected PSMC3 (*P* = 0.039). The mRNA abundance of succinate dehydrogenase complex, subunit A (SDHA) was higher in 5 d compared to 12 d LBW-Ala piglets (*P* = 0.009).

## Discussion

The SI of LBW piglets is developmentally and functionally compromised compared to NBW individuals [63, 64]. Oral Gln supplementation has been previously shown to be beneficial for the jejunal development and function of piglets around weaning [34, 35], however only few studies examined effects of Gln supplementation in piglets during the suckling phase [33, 38, 65]. While most studies have looked at weaned piglets, the present work is focused on the early suckling period, which to our knowledge has not been studied in this species. The jejunum is critical for the digestion of milk and absorption of nutrients. Therefore, in this work we focused on this section of the intestine and used a wide range of analytical methods to characterize potential effects of glutamine or alanine supplementation.

Our outgoing hypothesis was that the jejunal morphology and development of LBW compared to NBW male piglets benefits from Gln as compared to Ala supplementation. In addition, changes in jejunal AA profiles could provide insights into Gln jejunal metabolism and its potential role in improving LBW jejunal morphology and development.

### Comparison among supplementation groups

Oral Gln supplementation to LBW suckling piglets was not associated with changes in any of the jejunal parameters measured, when compared to LBW-Ala or NBW-Gln control groups, at 5 d or 12 d of life. In addition, no effects were observed when Gln supplementation was assessed within each age group irrespective of BiW. *In vitro* studies in intestinal porcine enterocytes have shown that media supplemented with 2 mM Gln increased FPSR [66] and cell growth [67], both of which were unaffected in this study. However, this is a concentration 2–8 times higher than that reported in piglet plasma [38, 68] and thus translation of results is difficult. Furthermore, more recent *in vitro* studies on intestinal porcine enterocytes have shown effects of Gln on ATP production and apoptosis [28, 69]. In LPS-challenged suckling piglets, Haynes et al. (2009) showed that oral Gln supplementation prevented endotoxin related villus atrophy [38]. *In vivo* studies investigating effects of Gln supplementation on intestinal physiology have been conducted in piglets at the end of the suckling period, but different parameters were evaluated [65]. However, the majority of studies were conducted in weaned piglets [29]. Their physiological conditions are very different from that of the suckling piglets used in this study as intestinal AA-metabolism, local immunity and cellular population are changing [15]. It has been reported that Gln supplementation during weaning improved growth performance and intestinal health by preventing villus atrophy and reducing antioxidative stress [33–35, 70]. Hsu et al. (2012) [71] observed increased tunica muscularis thickness in jejunum and ileum in weaned piglets upon Gln supplementation. In an infection study with pathogenic *E*. *coli* Gln supplementation of weaned piglets inhibited villus atrophy [72]. Thus, it appears that Gln may have a protective effect on the SI under stressful conditions such as infection [38, 72] and weaning [33–35, 70]. Another study reported that Gln supplementation had similar effects on growth performance and plasma concentration of TNF-alpha in weaned piglets as antibiotic treatment [36]. Although we observed in a companion study with the same experimental animals that plasma Gln concentrations were higher 2 h after oral Gln supplementation in the 5 d and 12 d old piglets compared to Ala supplementation [42], in jejunal digesta and tissue the FAA and PBAA Gln concentrations and that of its metabolite Glu were not different between the Ala and Gln piglets. This may indicate that the Gln dose was absorbed in the proximal SI (duodenum and/or proximal jejunum) [29].

It has been reported that excess Gln is stored in the skeletal muscle [73] and that skeletal muscle is one of the main locations of Gln synthesis [74]. Glutamine is released under stressful conditions such as starvation or infection from the skeletal muscle, and the synthesis of Gln increases under such conditions [74]. A companion paper, using the same animals as in this study, showed no difference in free Gln concentrations in the *M*. *longissimus dorsi* of LBW-Gln when compared to LBW-Ala or NBW-Gln groups, at 5 d or 12 d of life [75]. However, the concentration of Ala in *M*. *longissimus dorsi* in the Ala supplemented NBW and LBW piglets was higher than in the Gln littermates at 5 and 12 d of life. A study by Stoll et al. (1998) [76], using ^13^C labelled AAs in suckling pigs, showed that Gln has a negative portal balance, indicating that Gln is utilized intensively by the SI. Thus, our observations, together with FAA profiles from the duodenum of these animals that show higher Gln concentrations in LBW-Gln and NBW-Gln compared to their Ala birth weight companions (unpublished data) suggest, that the supplemental Gln is already absorbed in more proximal regions of the SI. Additionally, it seems that the skeletal muscle as a Gln storage tissue [73], may not be relevant within the 2 h time period between Gln administration and sampling in this study.

### Comparison between birth weight groups

A possible reason for the absence of differences in jejunal development between LBW-Gln and LBW-Ala or NBW-Gln piglets could be linked to the birth weight range of LBW piglets in this study (0.8–1.2 kg vs. 1.4–1.8 kg). In fact, the range of BiW reported for LBW piglets is rather wide [24, 64, 77]. Apparently, differences in intestinal development and function between low and normal BiW piglets, were reported mostly in piglets with much lower body weights than used here [77–79]. For example, Xu et al. (1994) observed reduced jejunal VH, CD, intestinal thickness, total DNA, RNA and protein content in very low birth weight piglets (0.59 ± 0.34 kg) at birth (prior to suckling) compared to normal BiW (1.32 ± 0.47 kg) littermates. Another study investigating newborn low birth weight piglets (0.83 ± 0.04 kg) and normal birth weight piglets (1.66 ± 0.07 kg) showed decreased length and weight of the SI, decreased VH:CD ratio and reduced expression of genes related to oxidative defense in low birth weight piglets [80]. In contrast, neither Wang et al. (2016) [79] nor Wiyaporn et al. (2013) [81] did observe differences in proximal jejunum VH, or CD between newborn un-suckled LBW piglets (0.81 ± 0.02 kg; and 0.88 ± 0.02 kg) compared to normal littermates (1.30 ± 0.03 kg; and 1.47 ± 0.03 kg). Similarly, small intestinal villus height, width and depth did not differ according to BiW (Huygelen et al., 2015). Additionally, Thongsong et al. (2019) [82], utilizing the same experimental piglets as Wiyaporn et al. (2013), did not find an effect of BiW on mRNA abundance of jejunal glucose, peptide and AA transporters including SLC7A8, which we determined in the present study. In the present study we did not determine jejunal parameters in newborn un-suckled piglets, thus it is not known whether the jejunal parameters measured differed at birth in our piglets. Interestingly, Wang et al. (2016) reported that in un-suckled low BiW piglets’ jejunal permeability and tight junction (OCLN) mRNA abundance were higher, and antioxidant scavenger (Gpx1, CAT) mRNA abundance was lower compared to normal BiW littermates. Yet by 3 d of life, the differences were no longer present. The absence of difference on mRNA level related to oxidative defense in our study might indicate that LBW were not challenged by additional oxidative stress. Also Huygelen et al. (2015), did not observe differences in SI cell proliferation and in intestinal barrier function between low and normal BiW piglets at birth and after 3, 10, and 28 d of suckling. These results suggest three possibilities, (1) that the intestinal parameters measured in our study do not differ between low and normal BiW piglets, or (2) that differences observed at birth prior to suckling might have already disappeared during postnatal development if the nutritional requirements of piglets are met as reviewed by Everaert et al. (2017). Thirdly, it cannot be excluded that low birth weight piglets surviving the first 3 days of life are more vital and less comparable to the very low birth weight piglets with compromised intestinal development, which leads to a bias of selection of these piglets.

### Comparison of piglet age groups

The development of the SI during the suckling phase is characterized by rapid growth, both on a macroscopic [10] and microscopic [77] scale. Several studies indicate that SI maturation is accompanied with changes in enterocyte metabolism [15, 26, 83] and nutrient absorption kinetics [84]. In the current study, jejunal morphological and immunological markers as well as nucleic acid, protein and AA profiles were compared between 5 d and 12 d old piglets, irrespective of the Suppl or BiW group. Morphologically, higher CD, VH, VW and TuM were observed in the 12 d compared to the 5 d group, consistent with previous studies conducted in sow-reared piglets [10, 19, 77, 85]. Longer and wider villi, as well as deeper crypts observed in 12 d compared to 5 d old piglets reflect an increased absorptive area. An increasing CD also indicates a higher crypt cell production and is an indicator for maturation of villous enterocytes [85]. The higher TuM observed in the older age group indicates jejunal cell proliferation and maturation. It should be noted that conflicting results in regard to the development of VH with age have been reported [19, 77, 86], which appear to be related to differences in piglet age, SI segment, villus atrophy, creep feed consumption and milk intake [12, 86]. Generally, the shape and length of the villi in the small intestine changes with weaning [12]. We observed lower staining of mixed mucins containing goblet cells in the villi and crypts and lower total number of crypt goblet cells in piglets of the 12 d compared to the 5 d group. Goblet cells containing different mucin types act as an innate defense mechanism, where the mucins protect the gastrointestinal tract by acting as a diffusion and micro-ecological barrier [87]. The observed decrease may suggest that at 12 d of age the mucosal barrier function built by mucins is changing due to immune system maturation, or may indicate changing luminal bacteria composition [88]. In addition, the abundance of IgA positive cells in the lamina propria was assessed. These IgA positive cells are B-cells that are derived from the antigen-specific IgA-committed B cells in Peyer’s patches, which migrate to the lamina propria and function as part of the innate immune defense [89]. Consistent with previous studies [90, 91] our results show that IgA positive cells were mainly located in the lamina propria and were detected only in the 12 d group. The abundance of IgA positive plasma cells have been shown to be influenced by age [90, 92], commensal microbiota [93], and diet [94]. Taken together, these results indicate that the jejunum of the piglets in this study matures morphologically and immunologically from 5 d to 12 d of age and neither differences in maturation due to BiW nor to AA supplementation were observed.

Multivariate analysis via block PLS-DA showed that jejunal FAA, PBAA and amino-metabolites were the only variable blocks significantly affected by piglet age. Univariate analysis of the individual variables within each block confirmed this observation, revealing altered jejunal concentrations for almost all of the individual and grouped FAA and PBAA when 12 d were compared to 5 d piglets. A subsequent, more stringent univariate analysis (volcano plot) was performed to identify highly significant variables contributing to the age group separation in these blocks. Identified were Pro and β-Alanine, which were lower in the 12 d animals compared to 5 d, whilst the opposite was observed for Cys and Carnosine. The importance of these AA and amino-metabolites for the age-dependent development of the porcine intestine are not fully understood. It has been previously reported [84] that the capacity to absorb AA per length unit of intestine decreases, as the total length of intestine increases, potentially explaining the decrease for several AA concentrations observed in the older piglet group used in this study. Moreover it was shown that AA metabolism in the jejunum of piglets changes within the different periods of the suckling phase [83]. Why this occurs is currently not understood, but it could be linked to differences in the intestinal microbiota [95], changes in cell structure and function, or in AA metabolism [83] and absorption [11, 96].

In the present study, lower jejunal protein, RNA and DNA concentrations and increased RNA to DNA ratio, a measure of ribosomal RNA content or protein synthetic capacity was observed in piglets of the 12 d compared to the 5 d group. The DNA concentration is a marker of cell number, and lower concentrations in the 12 d group indicates that the numbers of cells per mg of jejunal tissue is lower potentially explaining why the protein, RNA, FAA and PBAA concentrations are decreased. Why the cell number decreased from 5 d to 12 d is not fully understood, but it may be linked to reappearing rise in apoptosis, after an enhanced mitosis accompanied by a reduction of apoptosis during the first days after birth [97]. In addition it should not be overlooked, that intestinal cell turnover is affected by nutrition and specific nutrients [15]. Whilst protein synthetic capacity increased, there was no effect of age on FPSR or the ratio of protein to DNA. Thus, in terms of protein synthesis, there appears to be no effect of age in these very young piglets.

Furthermore, the mRNA abundance of genes related to Gln/Ala-uptake and metabolism and glutathione production was assessed. We observed that the mRNA abundance of one Gln (SLC1A5) and one Ala (SLC1A4 transcript variant 1) transporter, two enzymes involved in Gln metabolism (AST-1, GLUD-1, SDHA) and three involved in the glutathione synthesis pathway (GCL, GSS and GPX4) were lower in piglets from the 12 d compared to the 5 d group. The GCL encodes the rate-limiting enzyme for the glutathione production, whilst GSS encodes the enzyme involved in the subsequent step and our results may indicate that oxidative defense via glutathione production was lower in piglets from the 12 compared to 5 d group. This observation is similar as in a previous study which showed a downwards trend of GPX4 expression, an enzyme converting glutathione to glutathione-disulfide in the presence of radical oxygen species, in the jejunum of suckling piglets after the age of d 14 [98]. The mRNA abundance of antioxidative enzymes is not only dependent on the age of the individual piglet, but on the sampled tissue as well [99]. Thus, within the context of earlier studies on ontogenetic development of the jejunum in suckling piglets, the results from this study are consistent with an adequate physiological development independent of BiW or Gln supplementation.

## Conclusion

This study is the first to investigate the effect of oral Gln supplementation on jejunal development and AA profiles in suckling low and normal birth weight piglets. Results show that Gln as compared to Ala supplementation and BiW appears to have only small effects on the measured jejunal parameters, whereas the effect of age was significant. These novel findings suggest that oral Gln supplementation might not be an appropriate way to stimulate the development of jejunum in the suckling period. However, it is conceivable that Gln might be beneficial in a more challenging environment. Thus further research is warranted to investigate more proximal sections of the GIT, or cellular proliferation, microbial composition and the abundance of tight junction proteins during jejunal development.

## Supporting information

S1 File Supplementation procedure. (DOCX)

## Abbreviations:

AA: Amino Acid
Aad: α-Aminoadipic acid
Ala: Alanine
BiW: Birth weight
CD: Crypt depth
FAA: Free amino acids
FPSR: Fractional protein synthesis rate
Gln: Glutamine
LBW: Low birth weight
LSM: Least squares means
NBW: Normal birth weight
PBAA: Protein-bound amino acids
SE: Standard error
SI: Small intestine
Suppl: supplementation group
TAA: Total amino acids
TuM: Tunica muscularis thickness
VH: Villus height
VW: Villus width
