# Supplementary material for: Effects of oral glutamine supplementation on jejunal morphology, development, and amino acid profiles in male low birth weight suckling piglets
Source: PLoS One. 2022 Apr 27;17(4):e0267357. doi: 10.1371/journal.pone.0267357 (PMC9045636; doi:10.1371/journal.pone.0267357)
Supplement: S1 File — (DOCX) [file pone.0267357.s001.docx]

# S1 File. Supplementation procedure

The method used to supplement the piglets is suitable for experimental purposes only but ensures that the complete daily dose is ingested. The calculated respective amino acid amounts of supplemental amino acids were weighed into pre-weighed disposable syringes and amino acids were dissolved by adding 2 ml of water. Two trained staff members performed the supplementation. One of the two performed the supplementation while the other gently restrained the pig. The conus of the syringe was placed close to the basis of the pig’s tongue and the amino acid solution fluid was installed carefully squeezed out of the disposable syringe in the mouth of the piglet. The head of the animal was stretched gently to ensure that the introduced fluid was fully swallowed. Syringes were rinsed with 1 ml of water and the procedure of drenching was repeated as described (Li et al., 2022). During the study, emptied syringes were randomly collected, dried and weighed to determine if the amino acid dosage were completely given to the piglets. A previous study showed that drenching is not a stressful intervention for suckling piglets (Van Tichelen et al., 2021).

1. Li Z, Sciascia M, Görs S, Nguyen N, Rayatdoost Baghal F, Schregel J, Tuchscherer A, Zentek J, Metges CC (2022): Glutamine supplementation moderately affects growth, plasma metabolite and free amino acid patterns in neonatal low birthweight piglets. British Journal of Nutrition : Feb 11;1-11. doi: 10.1017/S0007114522000459. Online ahead of print.
2. Van Tichelen K, Prims S, Ayuso M, Van Kerschaver C, Vandaele M, Degroote J, Van Cruchten S, Michiels J, Van Ginneken C (2021): Handling Associated with Drenching Does Not Impact Survival and General Health of Low Birth Weight Piglets. Animals (Basel), 11. Doi: [10.3390/ani11020404](https://dx.doi.org/10.3390%2Fani11020404).

# S1 Table. Primer sequences

| Process | Gene Name |  |  | Reference | Forward | Reverse | Product Size | Age | Efficiency | mean Cq |
| --- | --- | --- | --- | --- | --- | --- | --- | --- | --- | --- |
|  | Full | Abbreviated name | Ref Seq No. |  |  |  |  |  |  |  |
| AA transporter | Solute Carrier Family 1 Member 4 Variant 1 | SLC1A4V1 | XM_013996100.2 | This study | ACGCAACCAGCTATGAAGAG | GGCTACTCCCAACACCAAG | 137 | 5 | 1.831 | 24.1 |
|  |  |  |  |  |  |  |  | 12 | 1.895 | 29.1 |
|  | Solute Carrier Family 1 Member 4 | SLC1A4 | XM_021087450.1 | This study | AGCAGGACAGATTTTCACCATC | CCACAGCCAGGATCAAAGAG | 149 | 5 | 1.859 | 29.6 |
|  |  |  |  |  |  |  |  | 12 | 1.851 | 29.9 |
|  | Solute Carrier Family 38 Member 3 | SLC38A3 | XM_003358448.4 | This study | CTTCCTACAGAAAAGCCCCAG | CGTTGCTAAGGTTGAACACTG | 92 | 5 | 1.883 | 31.3 |
|  |  |  |  |  |  |  |  | 12 | 1.860 | 32.0 |
|  | Solute Carrier Family 38 Member 4 | SLC38A4 | XM_021092580.1 | This study | AGAGCATCAGTGGAGAAAGTG | TTGACTTTCAGCATCTTCGTTTG | 104 | 5 | 1.913 | 33.0 |
|  |  |  |  |  |  |  |  | 12 | 1.865 | 34.0 |
|  | Solute-linked carrier family A1 member 5 | SLC1A5 | XM_003355984.4 | This study | GACGTCCTACTCGGAGAGG | ATGTTCATGCCCTCCACC | 78 | 5 | 1.877 | 28.8 |
|  |  |  |  |  |  |  |  | 12 | 1.903 | 29.8 |
|  | Solute Carrier Family 6 Member 19 | SLC6A19 | XM_003359855.4 | Yang et al. (2016) | CCGTTGATAAGCGTCAGGAT | CACAACAACTGCGAGAAGGA | 155 | 5 | 1.867 | 25.1 |
|  |  |  |  |  |  |  |  | 12 | 1.842 | 25.5 |
|  | Solute Carrier Family 7 Member 8 | SLC7A8 | XM_021099239.1 | This study | AATCTTCGTCTCACCCAAAGG | CATAACACAGGGCTCCTACAG | 110 | 5 | 1.842 | 24.8 |
|  |  |  |  |  |  |  |  | 12 | 1.815 | 26.7 |
| AA-metabolism/ Energy metabolism | Alanine aminotransferase | ALT | XM_013996392.2 | This study | TGAAGGAGAAGGTGCTAACG | CTCGGATGACCTCAGTGAAG | 150 | 5 | 1.776 | 32.5 |
|  |  |  |  |  |  |  |  | 12 | 1.750 | 33.5 |
|  | Glutaminase | GLS | XM_021076054.1 | This study | CAGTTTGATGCATTCATGTGG | CCAGATTTTGCAGGAAGACC | 78 | 5 | 1.919 | 27.1 |
|  |  |  |  |  |  |  |  | 12 | 1.870 | 24.2 |
|  | Aspartate aminotransferase 1 | AST 1 | NM_213927.1 | This study | CTTGCCCTTGGAGATGACAG | TCCATTGTACCATCGTGCTAAG | 120 | 5 | 1.718 | 28.0 |
|  |  |  |  |  |  |  |  | 12 | 1.715 | 29.9 |
|  | Aspartate aminotransferase 2 | AST 2 | NM_213928.1 | This study | TGGGTGAGAACAATGAGGTG | TTTCGGCAGGAAGACATCTC | 137 | 5 | 1.894 | 23.3 |
|  |  |  |  |  |  |  |  | 12 | 1.875 | 24.8 |
|  | Glutamate dehydrogenase | GLUD1 | NM_001244501.2 | This study | TCCTGGGAGGTTATCGAGG | ACTTCATCTACGCTCACATCAG | 101 | 5 | 1.686 | 29.5 |
|  | N-acetylglutamate synthase | NAGS | NM_001097520.1 | This study | ACTCTGTCTACGTCTCTGAGG | CCACAAACTTGTCTAGATACGGG | 93 | 5 | 1.852 | 27.9 |
|  |  |  |  |  |  |  |  | 12 | 1.859 | 28.9 |
|  | 26S protease regulatory subunit 6A | PSMC3 | XM_005660922.3 | This study | GCAAGTGTGCAGTGATCAAAA | TCTTTGTTCACACCCACCAA | 112 | 5 | 1.929 | 24.4 |
|  |  |  |  |  |  |  |  | 12 | 1.887 | 25.9 |
|  | Succinate dehydrogenase complex, subunit A | SDHA | XM_021076931.1 | Feng et al. (2010) | GCGGACAGAGCCTCAAGTT | CAGGAGATCCAAGGCAAAAT | 155 | 5 | 1.822 | 25.2 |
|  |  |  |  |  |  |  |  | 12 | 1.842 | 25.9 |
| Oxidative stress + Glutathione metabolism | Cysthathionine-gamma-Lyase | CGL | NM_001044585.1 | This study | CTTCATAGTAGGCTCCGTTT | ACTGAGCAACTGCCATTCCA | 142 | 5 | 1.932 | 25.2 |
|  |  |  |  |  |  |  |  | 12 | 1.893 | 26.2 |
|  | Glutamate cysteine ligase | GCL | XM_021098556.1 | Rasch et al. (2019) | TGATCCCCATCCTGAACTCT | CGGATGGTTTGCGATAAACT | 155 | 5 | 1.892 | 25.4 |
|  |  |  |  |  |  |  |  | 12 | 1.900 | 26.4 |
|  | Copper dependent superoxide dismutase | CuSOD | NM_001190422.1 | This study | GAGACCTGGGCAATGTGACT | CTGCCCAAGTCATCTGGTTT | 139 | 5 | 1.905 | 23.1 |
|  |  |  |  |  |  |  |  | 12 | 1.901 | 22.4 |
|  | Gamma-Glutamyl-Transferase | GGT | XM_021072100.1 | Rasch et al. (2019) | CAAAGGGTACAACTTCTCC | AGCCAAAGTAGAGGTTGAT | 338 | 5 | 1.847 | 24.7 |
|  |  |  |  |  |  |  |  | 12 | 1.844 | 25.8 |
|  | Glutathione peroxidase 1 | GPX1 | NM_214201.1 | This study | GGGACTACACCCAGATGAATG | CTGATGTCCAAACTGGTTGC | 92 | 5 | 1.775 | 22.5 |
|  |  |  |  |  |  |  |  | 12 | 1.817 | 28.1 |
|  | Glutathione peroxidase 2 | GPX2 | NM_001115136.1 | This study | GTGGTTCTTGGCTTCCCTT | CGTACTTGAGGCTGTTCAGG | 82 | 5 | 1.845 | 21.8 |
|  |  |  |  |  |  |  |  | 12 | 1.905 | 25.5 |
|  | Glutathione peroxidase 4 | GPX4 | NM_214407.1 | This study | ACGAATTCTCAGCCAAGGAC | CTCATTGAGAGGCCACATTG | 96 | 5 | 1.700 | 25.4 |
|  | Glutathione-disulfide Reductase | GSR1 | XM_003483635.4 | This study | TGGAGAATGCTGGCATAGAG | GGGTTTCCTACCAGGAACTG | 110 | 5 | 1.907 | 24.7 |
|  |  |  |  |  |  |  |  | 12 | 1.883 | 26.3 |
|  | Glutathione synthetase | GSS | NM_001244625.1 | Rasch et al. (2019) | GTGCTCAAGCCCCAGAGA | ATGAGGCTCTCTCCTCACTGTC | 100 | 5 | 1.932 | 27.3 |
|  |  |  |  |  |  |  |  | 12 | 1.914 | 28.1 |
|  | Glutathione S-transferases | GSTA | NM_213850.2 | This study | CCGTTATCTTCCTGCATTTG | GTCAGCCCTGCTCAGCTT | 82 | 5 | 1.930 | 22.7 |
|  |  |  |  |  |  |  |  | 12 | 1.890 | 22.7 |
|  | Heat Shock Protein 90kDa Alpha | HSPCB | XM_005666063.2 | This study | AGATCACCTGGCAGTCAAGC | CCGCGGATGAAGTTGAGGTA | 198 | 5 | 1.889 | 22.4 |
|  |  |  |  |  |  |  |  | 12 | 1.907 | 18.5 |
| Reference gene | Peptidylprolyl isomerase A | PPIA | NM_214353 | Rasch et al. (2019) | AGCACTGGGGAGAAAGGATT | AAAACTGGGAACCGTTTGTG | 220 | 5 | 1.895 | 18.7 |
|  |  |  |  |  |  |  |  | 12 | 1.895 | 21.3 |
|  | Ribosomal protein S18 | RPS 18 | NM_213940.1 | Rasch et al. (2019) | GGATGTGAAGGATGGGAAGTACA | TCCAAGTCTTCACGGAGTTTGTT | 72 | 5 | 1.887 | 22.8 |
|  |  |  |  |  |  |  |  | 12 | 1.887 | 21.0 |
|  | Actin beta | ACTB | XM_021086047.1 | Nygard et al. (2007) | CACGCCATCCTGCGTCTGGA | AGCACCGTGTTGGCGTAGAG | 100 | 5 | 1.915 | 18.6 |
|  |  |  |  |  |  |  |  | 12 | 1.740 | 25.8 |
|  | Topoisomerase 2-beta | TOP2B | NM_001258386.1 | This study | CCCAGTTGGCTGGATCTGTT | ATAACGAGGGCTTGCAGCAT | 182 | 5 | 1.907 | 25.9 |
|  |  |  |  |  |  |  |  | 12 | 1.901 | 25.5 |

**S1 Table References**

1. Yang C, Yang X, Lackeyram D, Rideout TC, Wang Z, Stoll B, et al. Expression of apical Na(+)-L-glutamine co-transport activity, B(0)-system neutral amino acid co-transporter (B(0)AT1) and angiotensin-converting enzyme 2 along the jejunal crypt-villus axis in young pigs fed a liquid formula. Amino acids. 2016;48(6):1491-508.

2. Feng XT, Xiong YZ, Qian H, Lei MG, Xu DQ, Ren ZQ. Selection of reference genes for gene expression studies in porcine skeletal muscle using SYBR green qPCR. J Biotechnol. 2010;150(3):288-93.

3. Rasch I, Gors S, Tuchscherer A, Htoo JK, Kuhla B, Metges CC. Substitution of Dietary Sulfur Amino Acids by DL-2-hydroxy-4-Methylthiobutyric Acid Increases Remethylation and Decreases Transsulfuration in Weaned Piglets. J Nutr. 2019;149(3):432-40.

4. Nygard AB, Jorgensen CB, Cirera S, Fredholm M. Selection of reference genes for gene expression studies in pig tissues using SYBR green qPCR. BMC Mol Biol. 2007;8:67.

# S2 Table. Number of jejunal IgA positive cells in low and normal birth weight male suckling piglets

|  |  | **Ala** | |  | **Gln** | |  | ***P* values^1^** |
| --- | --- | --- | --- | --- | --- | --- | --- | --- |
| **Item** | **Age ² (d)** | **LBW** | **NBW** |  | **LBW** | **NBW** | **SE** | **Suppl** |
| Area1 | 12 | 0.33 | 0.17 |  | 0.00 | 0.83 | 0.19 | 0.281 |
| Area2 | 12 | 0.83 | 0.33 |  | 0.83 | 0.17 | 0.15 | 0.586 |
| Area3 | 12 | 9.83 | 10.1 |  | 8.25 | 8.92 | 0.68 | 0.048 |

Values are LSM ± SE of IgA positive cells per 10000 μm^2^ of Lamina propria, the largest SE is shown; n = 12/group (5, 12 d).

^1^ANOVA F test. None of the other fixed factors (BiW or Age) nor the interactions of the fixed factors (Suppl x BiW; Suppl x Age; BiW x Age or Suppl x BiW x Age) were significant (*P* > 0.05).

² IgA positive cells not detectable on day 5.

Abbreviations: Area1 = villus; Area2 = end of villus/crypt mouth; Area 3 = Area beside the crypt region.

# S3 Table. Jejunal villi intraepithelial CD3 positive cells from suckling low and normal birth weight male piglets

|  |  | **Ala** | |  | **Gln** | |  |
| --- | --- | --- | --- | --- | --- | --- | --- |
| **Item** | **Age (d)** | **LBW** | **NBW** |  | **LBW** | **NBW** | **SE** |
| CD3 | 5 | 5.50 | 5.45 |  | 6.30 | 5.90 | 0.50 |
|  | 12 | 6.57 | 6.27 |  | 6.33 | 6.76 | 0.52 |

Values are LSM ± SE of CD3 positive cells per 10000 μm² of Lamina propria, the largest SE is shown; n = 12/group (5, 12d).

ANOVA F test. None of the fixed factors (Suppl, BiW or Age) or the interactions of the fixed factors (Suppl x BiW; Suppl x Age; BiW x Age or Suppl x BiW x Age) were significant (*P* > 0.05).

# S4 Table. Concentrations of jejunal free amino acids in suckling low and normal birth weight male piglets

|  |  | **Ala** | |  | **Gln** | |  | ***P* values^1^** | |
| --- | --- | --- | --- | --- | --- | --- | --- | --- | --- |
| **Item** | **Age (d)** | **LBW** | **NBW** |  | **LBW** | **NBW** | **SE** | **Age** | **BiW*Suppl*Age** |
| **IAA, µmol/g _(fresh matter)_** | | | | | | | | | |
| Arg | 5 | 2.70 | 2.02 |  | 2.42 | 2.65 | 0.30 | 0.003 | 0.081 |
|  | 12 | 1.62 | 1.79 |  | 2.02 | 1.86 | 0.31 |  |  |
| His | 5 | 1.07^e^ | 0.82 |  | 1.00 | 1.10 | 0.10 | 0.001 | 0.050 |
|  | 12 | 0.59^f^ | 0.66 |  | 0.75 | 0.70 | 0.10 |  |  |
| Ile | 5 | 1.53^e^ | 1.15 |  | 1.41 | 1.56 | 0.15 | 0.001 | 0.047 |
|  | 12 | 0.81^f^ | 0.90 |  | 1.04 | 0.92 | 0.16 |  |  |
| Leu | 5 | 3.51 | 2.59 |  | 3.18 | 3.55 | 0.38 | 0.01 | 0.057 |
|  | 12 | 2.01 | 2.23 |  | 2.55 | 2.28 | 0.39 |  |  |
| Lys | 5 | 3.00 | 2.18 |  | 2.60 | 2.99 | 0.33 | 0.051 | 0.056 |
|  | 12 | 1.82 | 2.01 |  | 2.34 | 2.18 | 0.34 |  |  |
| Met | 5 | 1.17 | 0.91 |  | 1.07 | 1.19 | 0.12 | 0.004 | 0.049 |
|  | 12 | 0.67 | 0.75 |  | 0.83 | 0.74 | 0.12 |  |  |
| Phe | 5 | 1.94 | 1.61 |  | 1.87 | 2.03 | 0.18 | 0.005 | 0.088 |
|  | 12 | 1.25 | 1.37 |  | 1.46 | 1.31 | 0.19 |  |  |
| Thr | 5 | 2.57^e^ | 1.95 |  | 2.28 | 2.59 | 0.25 | 0.003 | 0.040 |
|  | 12 | 1.44^f^ | 1.57 |  | 1.79 | 1.64 | 0.25 |  |  |
| Trp | 5 | 0.46 | 0.37 |  | 0.42 | 0.46 | 0.04 | 0.016 | 0.060 |
|  | 12 | 0.30 | 0.33 |  | 0.37 | 0.34 | 0.04 |  |  |
| Val | 5 | 2.53 | 1.87 |  | 2.25 | 2.53 | 0.26 | 0.006 | 0.037 |
|  | 12 | 1.39 | 1.56 |  | 1.83 | 1.63 | 0.26 |  |  |
| BCAA | 5 | 7.58 | 5.60 |  | 6.84 | 7.63 | 0.79 | 0.005 | 0.047 |
|  | 12 | 4.21 | 4.69 |  | 5.42 | 4.83 | 0.81 |  |  |
| Total IAA | 5 | 20.5 | 15.5 |  | 18.5 | 20.6 | 2.09 | 0.008 | 0.053 |
|  | 12 | 11.9 | 13.2 |  | 15.0 | 13.6 | 2.14 |  |  |
| **DAA, µmol/g _(fresh matter)_** | | | | | | | | | |
| Ala | 5 | 7.16^e^ | 5.97 |  | 6.32^e^ | 6.62^e^ | 0.46 | <0.001 | 0.066 |
|  | 12 | 3.89^f^ | 4.17 |  | 4.11^f^ | 3.71^f^ | 0.47 |  |  |
| Asn | 5 | 1.59 | 1.33 |  | 1.51 | 1.58 | 0.15 | 0.003 | 0.136 |
|  | 12 | 0.94 | 1.01 |  | 1.17 | 1.08 | 0.15 |  |  |
| Asp | 5 | 3.18 | 2.67 |  | 3.13 | 3.29^e^ | 0.23 | 0.002 | 0.025 |
|  | 12 | 2.24 | 2.55 |  | 2.53 | 2.23^f^ | 0.23 |  |  |
| Cys | 5 | 0.71^f^ | 0.54^f^ |  | 0.72^f^ | 0.67^f^ | 0.10 | <0.001 | 0.256 |
|  | 12 | 1.65^e^ | 1.56^e^ |  | 1.65^e^ | 1.33^e^ | 0.10 |  |  |
| Gln | 5 | 2.90^e^ | 2.30^b^ |  | 3.06 | 3.33^ea^ | 0.26 | <0.001 | 0.036 |
|  | 12 | 1.60^f^ | 1.83 |  | 2.13 | 1.95^f^ | 0.27 |  |  |
| Glu | 5 | 8.78 | 8.06 |  | 9.70^e^ | 9.02^e^ | 0.53 | <0.001 | 0.314 |
|  | 12 | 6.68 | 7.28 |  | 7.37^f^ | 6.70^f^ | 0.54 |  |  |
| Gly | 5 | 7.80 | 7.11^f^ |  | 7.50^f^ | 7.71 | 0.45 | <0.001 | 0.259 |
|  | 12 | 9.48 | 10.0^e^ |  | 9.84^e^ | 9.56 | 0.46 |  |  |
| Pro | 5 | 9.14^e^ | 7.27^e^ |  | 8.36^e^ | 8.37^e^ | 0.62 | <0.001 | 0.096 |
|  | 12 | 2.47^f^ | 2.83^f^ |  | 3.34^f^ | 2.91^f^ | 0.62 |  |  |
| Ser | 5 | 4.85^e^ | 3.81 |  | 4.43 | 4.89^e^ | 0.41 | <0.001 | 0.044 |
|  | 12 | 2.57^f^ | 2.81 |  | 3.15 | 2.87^f^ | 0.42 |  |  |
| Tyr | 5 | 1.82 | 1.46 |  | 1.72 | 1.82 | 0.17 | 0.010 | 0.092 |
|  | 12 | 1.19 | 1.31 |  | 1.40 | 1.27 | 0.17 |  |  |
| Total DAA | 5 | 48.0 | 40.7 |  | 46.4 | 47.3^e^ | 2.88 | <0.001 | 0.062 |
|  | 12 | 32.7 | 35.3 |  | 36.7 | 33.6^f^ | 2.92 |  |  |
| **Total AA** | 5 | 74.3 | 62.1 |  | 70.7 | 73.9 | 5.04 | 0.003 | 0.051 |
|  | 12 | 52.7 | 57.2 |  | 60.3 | 55.2 | 5.13 |  |  |

Values are LSM ± SE, the largest SE is shown; *n* = 12/group (5, 12 d). Only AA affected by AA supplementation, BiW or age are shown.

^a,b^ Labeled LSM in a row within one BiW group and one age-group without a common letter differ, *P* < 0.05 (Tukey-Kramer test).

^e,f^ Labeled LSM in a column within one combined Suppl - BiW group without a common letter differ, *P* < 0.05 (Tukey-Kramer test).

^1^ANOVA F test. BiW affected Cys (P < 0.05), Suppl affected Gln (P < 0.05); none of the interactions of the fixed factors (Suppl x BiW; Suppl x Age or BiW x Age) were significant (P > 0.05).

Abbreviations: AA, Amino acids; BCAA, Branched-chain amino acids; DAA, Dispensable amino acids IAA, Indispensable amino acids.

# S5 Table. Jejunal amino metabolites from suckling low and normal birth weight male piglets

|  |  | **Ala** | |  | **Gln** | |  | ***P* values^1^** | |
| --- | --- | --- | --- | --- | --- | --- | --- | --- | --- |
| **Item** | **Age (d)** | **LBW** | **NBW** |  | **LBW** | **NBW** | **SE** | **Age** | **BiW*Suppl*Age** |
| **Amino metabolites, µmol/g _(fresh matter)_** | | | | | | | | | |
| 3-MH | 5 | 0.010 | 0.008 |  | 0.012^e^ | 0.010 | 0.001 | 0.002 | 0.993 |
|  | 12 | 0.005 | 0.005 |  | 0.006^f^ | 0.006 | 0.001 |  |  |
| Aad | 5 | 0.225^e^ | 0.171 |  | 0.196^e^ | 0.238^e^ | 0.022 | <0.001 | 0.013 |
|  | 12 | 0.081^f^ | 0.079 |  | 0.092^f^ | 0.081^f^ | 0.023 |  |  |
| Abu | 5 | 0.021 | 0.021 |  | 0.026 | 0.023 | 0.005 | 0.008 | 0.953 |
|  | 12 | 0.035 | 0.031 |  | 0.036 | 0.029 | 0.005 |  |  |
| β-Alanine | 5 | 0.076^e^ | 0.069 |  | 0.087^e^ | 0.082^e^ | 0.006 | <0.001 | 0.818 |
|  | 12 | 0.047^f^ | 0.048 |  | 0.045^f^ | 0.044^f^ | 0.006 |  |  |
| Car | 5 | 0.000^e^ | 0.000^e^ |  | 0.000^e^ | 0.000^e^ | 0.002 | <0.001 | 0.691 |
|  | 12 | 0.027^f^ | 0.029^f^ |  | 0.027^f^ | 0.026^f^ | 0.002 |  |  |
| Cit | 5 | 0.173 | 0.200 |  | 0.188 | 0.232 | 0.022 | 0.644 | 0.273 |
|  | 12 | 0.192 | 0.207 |  | 0.230 | 0.201 | 0.023 |  |  |
| GABA | 5 | 0.120 | 0.125 |  | 0.135 | 0.125 | 0.012 | 0.008 | 0.687 |
|  | 12 | 0.098 | 0.097 |  | 0.115 | 0.086 | 0.012 |  |  |
| Hyp | 5 | 0.503^e^ | 0.557^e^ |  | 0.561^e^ | 0.480^e^ | 0.046 | <0.001 | 0.375 |
|  | 12 | 0.257^f^ | 0.283^f^ |  | 0.273^f^ | 0.266^f^ | 0.047 |  |  |
| Orn | 5 | 0.062 | 0.061 |  | 0.067 | 0.083 | 0.010 | 0.478 | 0.239 |
|  | 12 | 0.080 | 0.080 |  | 0.074 | 0.063 | 0.010 |  |  |
| Tau | 5 | 2.41 | 2.80 |  | 2.51 | 2.52 | 0.21 | 0.241 | 0.989 |
|  | 12 | 2.60 | 2.82 |  | 2.89 | 2.72 | 0.21 |  |  |

Values are LSM ± SE, the largest SE is shown; *n* = 12/group (5, 12 d). Only AA and metabolites which are affected by AA supplementation, BiW or age-groups are shown.

^e,f^Labeled LSM in a column within one combined treatment - BiW group without a common letter differ, *P* < 0.05 (Tukey-Kramer test).

^1^ANOVA F test. BiW x Suppl affected Aad (*P* < 0.05); none of the other fixed factors (Suppl or BiW) or the interactions of the fixed factors (Suppl x Age or BiW x Age) were significant (P > 0.05).

Abbreviations: 3-MH, 3 Methylhistidine; Aad, α-Aminoadipate; Abu, α-Aminobutyric acid; Car, Carnosine; Cit, Citrulline; GABA, γ-Aminobutyric acid; Hyp, Hydroxyprolin; Orn, Ornithine; Tau, Taurine.

# S6 Table. Volcano plot of jejunal variables analysed between 5 and 12 day old suckling piglets

| **Variable Block** | **Item** | **Fold change [Cohen´s d]** | **FDR** |
| --- | --- | --- | --- |
| Jejunal tissue PB AA | Ala | 1.08 | < 0.001 |
|  | Asp | 1.05 | < 0.001 |
|  | Cys | 1.23 | < 0.001 |
|  | Ile | 1.24 | < 0.001 |
|  | Leu | 1.01 | < 0.001 |
|  | Lys | 1.83 | < 0.001 |
|  | Phe | 1.04 | < 0.001 |
|  | Thr | 1.13 | < 0.001 |
|  | Tyr | 1.12 | < 0.001 |
|  | Val | 1.30 | < 0.001 |
| Jejunal tissue free AA | Cys | -1.74 | < 0.001 |
|  | Pro | 1.55 | < 0.001 |
| Jejunal tissue AA metabolites | Car | -1.62 | < 0.001 |
|  | β-Ala | 1.18 | < 0.001 |
| Jejunal tissue  Biochemical indices | Protein | 1.82 | < 0.001 |
|  |  |  |  |

FC: fold change relative to 12 d age group. Values with a positive sign indicate that larger values are present in the 5 d group. Values with a negative sign indicate that the corresponding value is larger in the 12 d group.

*P*-values were calculated from Tukey Kramer test.

Tier 1 - Block of variables

Tier 2- Item name

Only items with both fold-change ≥ 1.0 or  ≤  -1.0 and FDR ≤ 0.05 are shown.

# S7 Table. Jejunal protein bound amino acids from suckling low and normal birth weight male piglets at the age of 5 d and 12 d

|  |  | **Ala** | |  | **Gln** | |  | **P values^1^** |
| --- | --- | --- | --- | --- | --- | --- | --- | --- |
| **Item** | **Age (d)** | **LBW** | **NBW** |  | **LBW** | **NBW** | **SE** | **Age** |
| **IAA, µmol/g _(fresh matter)_** | | | | | | | | |
| Arg | 5 | 35.8^e^ | 34.5 |  | 36.4 | 34.7^e^ | 1.27 | <0.001 |
|  | 12 | 29.8^f^ | 30.1 |  | 31.1 | 28.4^f^ | 1.30 |  |
| His | 5 | 15.1 | 14.6 |  | 15.6 | 14.7 | 0.54 | 0.001 |
|  | 12 | 13.4 | 13.4 |  | 14.0 | 12.6 | 0.55 |  |
| Ile | 5 | 36.5^e^ | 34.7^e^ |  | 37.6^e^ | 35.5^e^ | 1.19 | 0.049 |
|  | 12 | 27.2^f^ | 27.7^f^ |  | 28.8^f^ | 26.2^f^ | 1.21 |  |
| Leu | 5 | 65.0^e^ | 61.8^e^ |  | 66.5^e^ | 63.1^e^ | 2.19 | <0.001 |
|  | 12 | 50.3^f^ | 51.6^f^ |  | 53.4^f^ | 49.2^f^ | 2.23 |  |
| Lys | 5 | 48.0^e^ | 46.1^e^ |  | 48.2^e^ | 45.9^e^ | 2.39 | <0.001 |
|  | 12 | 22.2^f^ | 24.5^f^ |  | 22.2^f^ | 18.6^f^ | 2.43 |  |
| Met | 5 | 13.8^e^ | 13.2 |  | 14.2^e^ | 13.5^e^ | 0.48 | <0.001 |
|  | 12 | 11.4^f^ | 11.7 |  | 12.0^f^ | 10.9^f^ | 0.49 |  |
| Phe | 5 | 27.6^e^ | 26.1^e^ |  | 28.2^e^ | 26.8^e^ | 0.94 | <0.001 |
|  | 12 | 21.1^f^ | 21.6^f^ |  | 22.4^f^ | 20.6^f^ | 0.96 |  |
| Thr | 5 | 38.6^e^ | 36.6^e^ |  | 39.3^e^ | 37.3^e^ | 1.34 | <0.001 |
|  | 12 | 28.9^f^ | 29.2^f^ |  | 30.7^f^ | 28.2^f^ | 1.36 |  |
| Trp | 5 | 8.21^e^ | 7.61 |  | 8.24 | 7.94^e^ | 0.31 | <0.001 |
|  | 12 | 6.65^f^ | 6.79 |  | 7.10 | 6.58^f^ | 0.31 |  |
| Val | 5 | 49.2^e^ | 46.6^e^ |  | 50.2^e^ | 47.7^e^ | 1.62 | <0.001 |
|  | 12 | 35.8^f^ | 36.5^f^ |  | 38.1^f^ | 34.8^f^ | 1.64 |  |
| BCAA | 5 | 151^e^ | 143^e^ |  | 154^e^ | 146^e^ | 4.97 | <0.001 |
|  | 12 | 113^f^ | 116^f^ |  | 120^f^ | 110^f^ | 5.05 |  |
| Total IAA | 5 | 352^e^ | 336^e^ |  | 362^e^ | 349^e^ | 11.7 | <0.001 |
|  | 12 | 281^f^ | 279^f^ |  | 294^f^ | 270^f^ | 11.9 |  |
| **DAA, µmol/g _(fresh matter)_** | | | | | | | | |
| Ala | 5 | 55.1^e^ | 53.3^e^ |  | 56.7^e^ | 53.8^e^ | 1.89 | <0.001 |
|  | 12 | 42.2^f^ | 42.5^f^ |  | 44.8^f^ | 40.8^f^ | 1.92 |  |
| Asn | 5 | 24.4^e^ | 23.4 |  | 25.2^e^ | 23.4^e^ | 0.85 | <0.001 |
|  | 12 | 19.8^f^ | 20.1 |  | 20.8^f^ | 19.0^f^ | 0.87 |  |
| Asp | 5 | 35.1^e^ | 33.1^e^ |  | 35.5^e^ | 36.5^e^ | 1.57 | <0.001 |
|  | 12 | 24.5^f^ | 24.7^f^ |  | 27.2^f^ | 24.6^f^ | 1.60 |  |
| Cys | 5 | 8.63^e^ | 7.48^e^ |  | 8.63^e^ | 8.15^e^ | 0.48 | <0.001 |
|  | 12 | 4.55^f^ | 4.63^f^ |  | 5.22^f^ | 5.05^f^ | 0.49 |  |
| Gln | 5 | 28.5 | 27.3 |  | 29.2 | 27.7 | 1.00 | <0.001 |
|  | 12 | 24.3 | 24.4 |  | 25.6 | 23.4 | 1.01 |  |
| Glu | 5 | 50.8^e^ | 48.3^e^ |  | 51.4^e^ | 49.7^e^ | 1.84 | <0.001 |
|  | 12 | 38.5^f^ | 39.5^f^ |  | 41.5^f^ | 38.0^f^ | 1.87 |  |
| Gly | 5 | 52.0 | 49.4 |  | 52.2 | 49.8^e^ | 1.95 | <0.001 |
|  | 12 | 43.5 | 43.4 |  | 44.5 | 41.3^f^ | 1.98 |  |
| Pro | 5 | 26.0 | 25.3 |  | 31.4 | 31.2 | 2.43 | 0.173 |
|  | 12 | 27.6 | 23.7 |  | 26.4 | 24.0 | 2.48 |  |
| Ser | 5 | 48.1^e^ | 45.7^e^ |  | 48.7^e^ | 46.5^e^ | 1.74 | <0.001 |
|  | 12 | 38.0^f^ | 38.0^f^ |  | 39.6^f^ | 36.9^f^ | 1.77 |  |
| Tyr | 5 | 23.3^e^ | 22.0^e^ |  | 23.7^e^ | 22.5^e^ | 0.82 | <0.001 |
|  | 12 | 17.4^f^ | 17.4^f^ |  | 18.4^f^ | 16.9^f^ | 0.83 |  |
| Total DAA | 5 | 352^e^ | 336^e^ |  | 362^e^ | 349^e^ | 11.7 | <0.001 |
|  | 12 | 281^f^ | 279^f^ |  | 294^f^ | 270^f^ | 11.9 |  |
| **Total AA** | 5 | 684^e^ | 651^e^ |  | 701^e^ | 670^e^ | 23.0 | <0.001 |
|  | 12 | 519^f^ | 524^f^ |  | 545^f^ | 498^f^ | 23.0 |  |

Values are LSM ± SE, the largest SE is shown; *n* = 12/group (5, 12 d); Only AA and AA ratios which are affected by AA supplementation, BiW or age are shown.

^e,f^Labeled LSM in a column between one Suppl - birth weight group without a common letter differ, *P* < 0.05 (Tukey-Kramer test).

^1^ANOVA F test. BiW affected Asn, and Ile (*P* < 0.05); neither the other fixed factor (Suppl) nor the interactions of the fixed factors (Suppl x BiW; Suppl x Age; BiW x Age or Suppl x BiW x Age) were significant (*P* > 0.05).

Abbreviations: AA, Amino acids; BCAA, Branched-chain amino acids; DAA, Dispensable amino acids IAA, Indispensable amino acids.

# S8 Table. Jejunal digesta free amino acid concentrations from suckling low and normal birth weight male piglets

|  |  | **Ala** | |  | **Gln** | |  | ***P* values^1^** |
| --- | --- | --- | --- | --- | --- | --- | --- | --- |
| **Item** | **Age (d)** | **LBW** | **NBW** |  | **LBW** | **NBW** | **SE** | **Age** |
| **IAA, µmol/g _(DM jejunal digesta)_** | | | | | | | | |
| Arg | 5 | 15.9 | 13.5 |  | 16.5 | 20.3 | 2.95 | 0.063 |
|  | 12 | 14.7 | 15.4 |  | 10.6 | 10.7 | 2.54 |  |
| His | 5 | 7.99 | 5.69 |  | 6.40 | 9.14 | 1.72 | 0.291 |
|  | 12 | 6.25 | 7.04 |  | 4.66 | 5.43 | 1.54 |  |
| Ile | 5 | 14.3 | 10.3 |  | 12.8 | 18.4 | 2.97 | 0.100 |
|  | 12 | 11.3 | 11.8 |  | 7.20 | 9.80 | 2.66 |  |
| Leu | 5 | 29.9 | 22.1 |  | 26.9 | 38.8 | 5.92 | 0.134 |
|  | 12 | 24.0 | 26.8 |  | 18.9 | 22.5 | 5.18 |  |
| Lys | 5 | 44.2 | 33.1 |  | 35.4 | 47.2 | 8.22 | 0.975 |
|  | 12 | 47.7 | 43.6 |  | 29.7 | 39.7 | 7.21 |  |
| Met | 5 | 7.52 | 4.99 |  | 6.51 | 8.53 | 1.54 | 0.038 |
|  | 12 | 4.55 | 4.75 |  | 3.20 | 3.81 | 1.40 |  |
| Phe | 5 | 14.6 | 11.2 |  | 14.3 | 18.2 | 2.97 | 0.207 |
|  | 12 | 12.5 | 14.1 |  | 9.36 | 11.0 | 2.63 |  |
| Thr | 5 | 9.82 | 9.06 |  | 9.09 | 17.3 | 2.87 | 0.666 |
|  | 12 | 11.1 | 12.6 |  | 7.42 | 10.5 | 2.54 |  |
| Trp | 5 | 2.99 | 2.26 |  | 2.83 | 4.10 | 0.71 | 0.552 |
|  | 12 | 2.93 | 3.32 |  | 2.23 | 2.42 | 0.63 |  |
| Val | 5 | 12.0 | 11.7 |  | 12.4 | 23.1 | 3.92 | 0.602 |
|  | 12 | 13.6 | 16.1 |  | 10.1 | 13.2 | 3.48 |  |
| BCAA | 5 | 56.2 | 44.2 |  | 52.1 | 80.2 | 12.6 | 0.213 |
|  | 12 | 49.0 | 54.8 |  | 36.2 | 45.5 | 11.2 |  |
| Total IAA | 5 | 160 | 124 |  | 143 | 205 | 31.1 | 0.296 |
|  | 12 | 149 | 156 |  | 103 | 129 | 27.4 |  |
| **DAA, µmol/g _(DM jejunal digesta)_** | | | | | | | | |
| Ala | 5 | 22.6 | 20.2 |  | 18.5 | 30.5 | 4.65 | 0.437 |
|  | 12 | 23.8 | 24.1 |  | 15.3 | 18.5 | 4.07 |  |
| Asn | 5 | 7.88 | 6.69 |  | 6.46 | 12.1 | 2.04 | 0.490 |
|  | 12 | 8.24 | 8.01 |  | 5.55 | 7.06 | 1.81 |  |
| Asp | 5 | 8.41 | 8.38 |  | 8.12 | 19.4 | 3.03 | 0.351 |
|  | 12 | 13.9 | 15.6 |  | 9.04 | 13.7 | 2.65 |  |
| Cys | 5 | 2.19 | 2.06 |  | 0.761 | 2.96 | 0.648 | 0.15 |
|  | 12 | 1.33 | 1.67 |  | 0.842 | 1.53 | 0.565 |  |
| Gln | 5 | 27.6 | 20.7 |  | 24.7 | 31.7 | 5.42 | 0.066 |
|  | 12 | 20.4 | 19.2 |  | 13.5 | 18.6 | 4.87 |  |
| Glu | 5 | 30.0 | 24.2 |  | 21.5 | 47.0 | 7.16 | 0.761 |
|  | 12 | 32.9 | 33.1 |  | 19.8 | 31.1 | 6.23 |  |
| Gly | 5 | 14.8 | 18.0 |  | 15.1 | 27.0 | 5.47 | 0.104 |
|  | 12 | 29.7 | 27.1 |  | 20.2 | 27.7 | 4.94 |  |
| Pro | 5 | 19.2 | 18.0 |  | 18.9 | 29.1 | 5.70 | 0.343 |
|  | 12 | 19.3 | 17.7 |  | 12.5 | 18.3 | 5.12 |  |
| Ser | 5 | 12.2 | 13.4 |  | 11.2 | 23.0 | 3.75 | 0.706 |
|  | 12 | 15.2 | 16.3 |  | 10.2 | 14.0 | 3.31 |  |
| Tyr | 5 | 21.2 | 14.6 |  | 18.9 | 20.4 | 3.05 | 0.015 |
|  | 12 | 14.2 | 14.2 |  | 10.1 | 12.2 | 2.72 |  |
| Total DAA | 5 | 163 | 145 |  | 143 | 243 | 36.7 | 0.608 |
|  | 12 | 179 | 177 |  | 118 | 163 | 32.7 |  |
| **Total AA** | 5 | 333 | 277 |  | 295 | 461 | 69.8 | 0.506 |
|  | 12 | 342 | 346 |  | 232 | 306 | 62.0 |  |

Values are LSM ± SE, the largest SE is shown; n = 12/group (5, 12 d). As the jejunum was not filled with digesta no further analysis were performed and therefore group size deviated from n = 12. 5 d LBW-Gln, and 5 d NBW-Gln *n* = 11; 5 d LBW-Ala *n* = 9*.*

^1^ANOVA F test. BiW affected Asp, and Ser (*P* < 0.05), Suppl x Age affected Arg, and Asp (*P* < 0.05), Suppl x BiW affected Glu, (*P* < 0.05); neither the other fixed factor (Suppl) nor the interactions of the fixed factors (BiW x Age or Suppl x BiW x Age) were significant (*P* > 0.05).

Abbreviations: AA, Amino acids; BCAA, Branched-chain amino acids; DAA, Dispensable amino acids; DM, Dry matter; IAA, Indispensable amino acids.

# S9 Table. Jejunal digesta amino metabolites concentrations from suckling low and normal birth weight male piglets

|  |  | **Ala** | |  | **Gln** | |  | ***P* values^1^** |
| --- | --- | --- | --- | --- | --- | --- | --- | --- |
| **Item** | **Age (d)** | **LBW** | **NBW** |  | **LBW** | **NBW** | **SE** | **Age** |
| **Amino metabolites, µmol/ g _(DM jejunal digesta)_** | | | | | | | | |
| Aad | 5 | 0.192 | 0.196^b^ |  | 0.210^d^ | 0.487^ace^ | 0.065 | 0.004 |
|  | 12 | 0.114 | 0.114 |  | 0.110 | 0.108^f^ | 0.058 |  |
| Abu | 5 | 0.122 | 0.157 |  | 0.094 | 0.103 | 0.035 | 0.024 |
|  | 12 | 0.074 | 0.040 |  | 0.051 | 0.040 | 0.031 |  |
| β-Alanine | 5 | 0.057 | 0.101 |  | 0.105 | 0.185 | 0.040 | 0.027 |
|  | 12 | 0.055 | 0.043 |  | 0.033 | 0.027 | 0.040 |  |
| Cit | 5 | 3.35 | 1.85 |  | 2.00 | 4.71 | 1.27 | 0.676 |
|  | 12 | 3.30 | 2.60 |  | 1.97 | 2.44 | 1.13 |  |
| GABA | 5 | 0.072 | 0.152 |  | 0.352 | 0.387 | 0.208 | 0.202 |
|  | 12 | 0.364 | 0.280 |  | 0.567 | 0.658 | 0.189 |  |
| Orn | 5 | 2.12 | 1.27 |  | 0.840 | 1.93 | 1.31 | 0.005 |
|  | 12 | 3.38 | 3.72 |  | 2.44 | 6.12 | 1.13 |  |

Values are LSM ± SE, the largest SE is shown; n = 12/group (5, 12 d). As the jejunum was not filled with digesta no further analysis were performed and therefore group size deviated from n = 12. 5 d LBW-Gln, and 5 d NBW-Gln *n* = 11; 5 d LBW-Ala *n* = 9*.*

^a,b^Labeled LSM in a row within one BiW group and one age group without a common letter differ, *P* < 0.05 (Tukey-Kramer test).

^c,d^Labeled LSM in a row within one supplementation group and one age group without a common letter differ, *P* < 0.05 (Tukey-Kramer test).

^e,f^Labeled LSM in a column within one combined treatment-BiW group without a common letter differ, *P* < 0.05 (Tukey-Kramer test).

^1^ANOVA F test. Suppl affected GABA (*P* < 0.05); neither the other fixed factor (BiW) or the interactions of the fixed factors (Suppl x BiW; Suppl x Age; BiW x Age or Suppl x BiW x Age) were significant (*P* > 0.05).

Abbreviations: Aad, α-Aminoadipate; Abu, α-Aminobutyric acid; Cit, Citrulline; DM, Dry matter; GABA, γ- Aminobutyric acid; Orn; Ornithine.

# S10 Table. Jejunal protein bound amino acid concentrations from suckling low and normal birth weight male piglets

|  |  | **Ala** | |  | **Gln** | |  | ***P* values^1^** |
| --- | --- | --- | --- | --- | --- | --- | --- | --- |
| **Item** | **Age (d)** | **LBW** | **NBW** |  | **LBW** | **NBW** | **SE** | **Age** |
| **IAA, µmol/g _(DM jejunal digesta)_** | | | | | | | | |
| Arg | 5 | 434 | 428 |  | 396 | 525 | 82.6 | 0.176 |
|  | 12 | 528 | 622 |  | 470 | 524 | 74.0 |  |
| His | 5 | 320 | 268 |  | 274 | 325 | 62.5 | 0.793 |
|  | 12 | 297 | 319 |  | 247 | 372 | 55.3 |  |
| Ile | 5 | 770 | 663 |  | 665 | 825 | 92.2 | 0.463 |
|  | 12 | 707 | 755 |  | 581 | 669 | 82.5 |  |
| Leu | 5 | 1176 | 1038 |  | 1055 | 1327 | 169 | 0.922 |
|  | 12 | 1134 | 1339 |  | 1029 | 1140 | 149 |  |
| Lys | 5 | 934 | 809^f^ |  | 836 | 1067 | 164 | <0.001 |
|  | 12 | 1439 | 1512^e^ |  | 1250 | 1410 | 142 |  |
| Met | 5 | 238 | 179 |  | 193 | 245 | 34.2 | 0.083 |
|  | 12 | 169 | 179 |  | 136 | 175 | 30.8 |  |
| Phe | 5 | 478 | 424 |  | 438 | 568 | 74.8 | 0.783 |
|  | 12 | 488 | 566 |  | 427 | 487 | 65.9 |  |
| Thr | 5 | 774 | 700 |  | 673 | 858 | 113 | 0.759 |
|  | 12 | 790 | 880 |  | 651 | 796 | 102 |  |
| Trp | 5 | 131 | 128 |  | 125 | 155 | 22.7 | 0.567 |
|  | 12 | 144 | 181 |  | 123 | 135 | 20.7 |  |
| Val | 5 | 1014 | 905 |  | 895 | 1138 | 139 | 0.759 |
|  | 12 | 959 | 1101 |  | 833 | 930 | 124 |  |
| BCAA | 5 | 2959 | 2606 |  | 2615 | 3290 | 397 | 0.807 |
|  | 12 | 2800 | 3196 |  | 2443 | 2738 | 353 |  |
| Total IAA | 5 | 6288 | 5544 |  | 5566 | 7047 | 895 | 0.444 |
|  | 12 | 6662 | 7464 |  | 5731 | 6623 | 792 |  |
| **DAA, µmol/g _(DM jejunal digesta)_** | | | | | | | | |
| Ala | 5 | 898 | 858 |  | 797 | 1047 | 145 | 0.257 |
|  | 12 | 1036 | 1189 |  | 899 | 966 | 128 |  |
| Asn | 5 | 567 | 493 |  | 486 | 592 | 68.0 | 0.813 |
|  | 12 | 582 | 599 |  | 502 | 502 | 59.9 |  |
| Asp | 5 | 512 | 498 |  | 491 | 740 | 121 | 0.07 |
|  | 12 | 726 | 871 |  | 624 | 718 | 108 |  |
| Cys | 5 | 177 | 194 |  | 142 | 185 | 48.9 | 0.058 |
|  | 12 | 235 | 328 |  | 224 | 239 | 44.6 |  |
| Gln | 5 | 1148 | 863 |  | 911 | 996 | 106 | 0.002 |
|  | 12 | 831 | 750 |  | 717 | 771 | 91.4 |  |
| Glu | 5 | 1430 | 1145 |  | 1184 | 1545 | 200 | 0.973 |
|  | 12 | 1424 | 1461 |  | 1091 | 1350 | 180 |  |
| Gly | 5 | 756 | 788 |  | 686 | 979 | 157 | 0.195 |
|  | 12 | 1011 | 1134 |  | 831 | 944 | 143 |  |
| Pro | 5 | 1645 | 1104 |  | 1262 | 1393 | 174 | 0.018 |
|  | 12 | 1011 | 1038 |  | 881 | 1018 | 158 |  |
| Ser | 5 | 1191 | 1045 |  | 995 | 1167 | 139 | 0.934 |
|  | 12 | 1162 | 1206 |  | 1000 | 1067 | 125 |  |
| Tyr | 5 | 474 | 404 |  | 425 | 504 | 57.7 | 0.388 |
|  | 12 | 414 | 465 |  | 370 | 410 | 51.0 |  |
| Total DAA | 5 | 8805 | 7400 |  | 7386 | 9155 | 1055 | 0.967 |
|  | 12 | 8441 | 9049 |  | 7137 | 7984 | 943 |  |
| **Total AA** | 5 | 15081 | 12937 |  | 12943 | 16189 | 1928 | 0.744 |
|  | 12 | 15103 | 16513 |  | 12855 | 14591 | 1716 |  |

Values are LSM ± SE, the largest SE is shown; *n* = 12/group (5, 12 d). As the jejunum was not filled with digesta no further analysis were performed and therefore group size deviated from n = 12. 5 d LBW-Gln, and 5 d NBW-Gln *n* = 11; 5 d LBW-Ala *n* = 9*.*

^e,f^Labeled LSM in a column between one treatment-birth weight group without a common letter differ, *P* < 0.05 (Tukey-Kramer test).

^1^ANOVA *F* test. Age x Suppl affected Pro (*P* < 0.05), none of the other fixed factors (Suppl or BiW) or the interactions of the fixed factors (Suppl x BiW; Suppl x Age; BiW x Age or Suppl x BiW x Age) were significant (P > 0.05).

Abbreviations: AA, Amino acids; BCAA, Branched-chain amino acids; DAA, Dispensable amino acids; DM, Dry matter; IAA, Indispensable amino acids.

# S11 Table. Jejunal mRNA abundance from suckling low and normal birth weight male piglets

|  |  | **Ala** | |  | **Gln** | |  | ***P* values^1^** |  |
| --- | --- | --- | --- | --- | --- | --- | --- | --- | --- |
| **Function** | **Age (d)** | **LBW** | **NBW** |  | **LBW** | **NBW** | **SE** | **Age** |  |
| **AA transporters** | | | | | | | | | |
| SLC1A4 | 5 | 0.08 | 0.09 |  | 0.15 | 0.12 | 0.09 | 0.055 |  |
|  | 12 | 0.07 | -0.12 |  | -0.05 | -0.05 | 0.10 |  |  |
| SLC1A5 | 5 | 0.06 | 0.04 |  | 0.21 | 0.13 | 0.07 | 0.028 |  |
|  | 12 | 0.07 | -0.02 |  | -0.09 | 0.01 | 0.08 |  |  |
| **AA metabolism** | | | | | | | | | |
| AST-2 | 5 | 0.22 | 0.06 |  | 0.13 | 0.01 | 0.07 | 0.039 |  |
|  | 12 | -0.0004 | -0.11 |  | 0.03 | 0.002 | 0.07 |  |  |
| **Antioxidative defense** | | | | | | | | | |
| PSMC3 | 5 | 0.30 | 0.04 |  | 0.10 | -0.005 | 0.08 | 0.094 |  |
|  | 12 | -0.04 | 0.004 |  | -0.03 | -0.02 | 0.08 |  |  |
| SDHA | 5 | 0.26^e^ | 0.15 |  | 0.22 | 0.12 | 0.06 | <0.001 |  |
|  | 12 | -0.07^f^ | -0.05 |  | 0.05 | -0.01 | 0.06 |  |  |
| CGL | 5 | 0.11 | 0.11 |  | 0.09 | 0.07 | 0.07 | 0.056 |  |
|  | 12 | 0.003 | 0.06 |  | -0.12 | -0.05 | 0.07 |  |  |
| GCL | 5 | 0.09 | 0.11 |  | 0.12 | 0.06 | 0.06 | 0.026 |  |
|  | 12 | -0.07 | -0.01 |  | 0.04 | 0.01 | 0.06 |  |  |
| GSS | 5 | 0.12 | 0.08 |  | 0.13 | 0.06 | 0.06 | 0.017 |  |
|  | 12 | 0.01 | -0.10 |  | 0.03 | 0.002 | 0.06 |  |  |

Values are LSM ± SE in arbitrary units and normalized to the most stable reference. The largest SE is shown; *n* = 12/group (5, 12 d). As for single samples mRNA abundance of selected targets was below the rate of quantification group size deviated from n = 12.

GPX-1 and GPX-2: 5 d LBW-Gln *n* = 11, and 5 d LBW-Ala *n* = 10

GSS: 12 d NBW-Gln *n* = 11

SLC1A4, SLC1A5, AST-1, SLC1A4V1: d 12 NBW Ala, d 12 NBW Gln, d 12 LBW Gln *n* = 11

^e,f^ Labeled LSM in a column between one Suppl - birth weight group without a common letter differ, *P* < 0.05 (Tukey-Kramer test).

^1^ ANOVA F test. BiW affected AST-2 (*P* = 0.020), BiW x Age interaction affected PSMC3 (*P* = 0.039); neither the other fixed factor (Suppl) or the interactions of the fixed factors (Suppl x BiW; Suppl x Age or Suppl x BiW x Age) were significant (*P* > 0.05). There was no significant effect of the fixed factors (Suppl, BiW or Age) or their interactions (Suppl x BiW; Suppl x Age; BiW x Age or Suppl x BiW x Age) (*P* > 0.05) on the mRNA abundances of genes related to amino acid transport: SLC6A19, SLC7A8,SLC1A4V1; Amino acid metabolism: AST-1, GLS, GLUD^2^, NAGS; and antioxidative defense: CuSOD, GGT, GPX-1, GPX-2, GPX-4, GSR, GSTA, HSPCB.

^2^ GLUD and GPX-4 were only quantified in the 5 d group because transcript abundance was too low in the 12 d group.

# S1 Figure. Sample plots of block PLS-DA


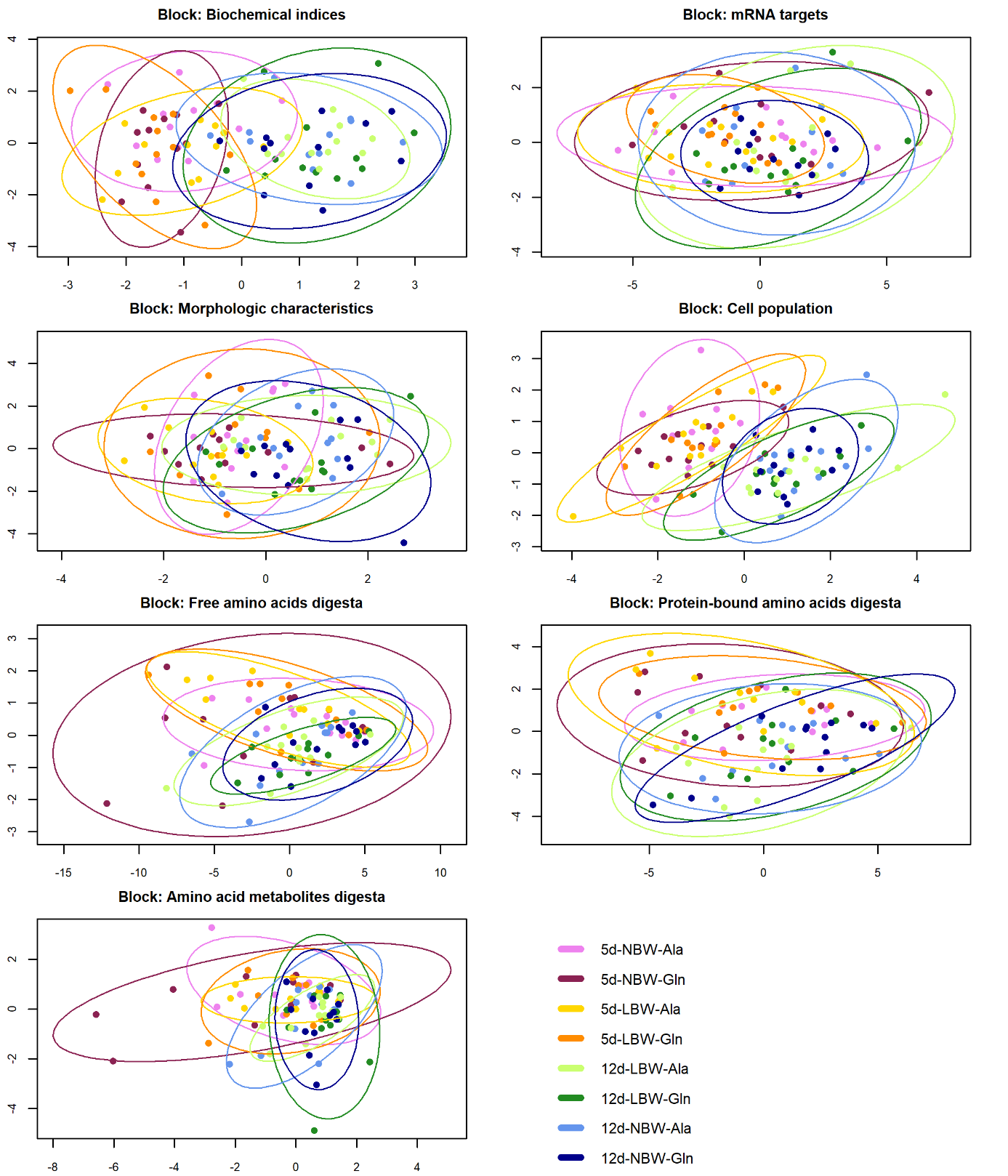
Sample plots of the block PLS-DA of all 143 jejunal variables assigned to ten variable groups (‘blocks’) and measured in 96 samples. Shown are the sample plots for the seven blocks with the best discriminatory ability: Biochemical indices, mRNA targets, Morphologic characteristics, Cell population, Free amino acids digesta, Protein-bound amino acids digesta, Amino acid metabolites digesta. The colours indicate the eight experimental groups of the 3-factorial crossed design (birth weight: LBW/NBW, supplementation: Ala/Gln and age group: 5 d/12 d) and highlight the main comparison of the two age groups (reddish: 5 d; bluegreen: 12 d).
